# Dunce Phosphodiesterase Acts as a Checkpoint for Drosophila Long-Term Memory in a Pair of Serotonergic Neurons

**DOI:** 10.1016/j.neuron.2018.03.032

**Published:** 2018-04-18

**Authors:** Lisa Scheunemann, Pierre-Yves Plaçais, Yann Dromard, Martin Schwärzel, Thomas Preat

**Affiliations:** 1 Genes and Dynamics of Memory Systems, Brain Plasticity Unit, ESPCI Paris, PSL Research University, CNRS, 10 rue Vauquelin, 75005 Paris, France; 2 Freie Universität Berlin, Department of Biology/Neurobiology, Königin-Luise Str. 28-30, Berlin 14195, Germany

**Keywords:** long-term memory, *Drosophila melanogaster*, serotonin, dopamine, phosphodiesterase, oscillations, mushroom body, cAMP, brain imaging, peduncle

## Abstract

A key function of the brain is to filter essential information and store it in the form of stable, long-term memory (LTM). We demonstrate here that the Dunce (Dnc) phosphodiesterase, an important enzyme that degrades cAMP, acts as a molecular switch that controls LTM formation in Drosophila. We show that, during LTM formation, Dnc is inhibited in the SPN, a pair of newly characterized serotonergic neurons, which stimulates the cAMP/PKA pathway. As a consequence, the SPN activates downstream dopaminergic neurons, opening the gate for LTM formation in the olfactory memory center, the mushroom body. Strikingly, transient inhibition of Dnc in the SPN by RNAi was sufficient to induce LTM formation with a training protocol that normally generates only short-lived memory. Thus, Dnc activity in the SPN acts as a memory checkpoint to guarantee that only the most relevant learned experiences are consolidated into stable memory.

## Highlights

• Dunce phosphodiesterase is a default inhibitor of long-term memory (LTM) formation
• Dunce acts in a pair of newly identified serotonergic projection neurons
• These serotonergic neurons control the activity of LTM-gating dopaminergic neurons

## Teaser

Long-term memory is executed only for selected experiences through regulatory mechanisms that are poorly characterized. Scheunemann et al. identify the phosphodiesterase Dunce acting in a pair of serotonergic neurons as a default inhibitor of long-term memory formation in Drosophila.

## Introduction

The brain constantly categorizes our environment and sorts out important information from “noise” or less relevant input (Petersen and Posner, 2012). Accordingly, most stimuli are meaningless for the brain; some are processed and generate a memory that is short lived; and, finally, a small proportion of information stands out and is consolidated into a long-lasting memory that remains available for adaptive behavior. These discriminative mechanisms are largely involved in context-dependent decision making and are crucial for the fitness and survival of any organism. This implies a strong evolutionary reinforcement of mechanisms that link information selection and memory mechanisms in the brain. However, the neuronal correlates that evaluate a presented stimulus and the functional link to memory processes are largely unknown.

During associative olfactory learning, an avoidance or attraction behavior to a previous neutral stimulus is generated by temporal pairing with a negative or positive unconditioned stimulus. A hallmark of memory storage across phyla is that spaced training, which signifies the facing of the same experience repeatedly with resting intervals, produces stronger and longer lasting memory than a single training (Cepeda et al., 2006, Pinsker et al., 1973). Thus, temporal contiguity between the neutral stimulus and the unconditioned stimulus is not the only important feature in associative learning; the global context of the association is also crucial. Brain mechanisms to filter information might, therefore, influence associative memory processes more directly than previously recognized (Ungless, 2004). The importance of suppressor mechanisms in these processes has been suggested, representing so-called “memory checkpoints” that restrict consolidation to a given context (Abel et al., 1998). Phosphodiesterases (PDEs) are enzymes that degrade cyclic AMP (cAMP), a second messenger that propagates cellular activation in most neurons by modulating the activity of protein kinase A (PKA) (Berke and Wu, 2002). PDEs, therefore, represent a potential memory checkpoint. Indeed, PDE inhibitors have been repeatedly shown to improve memory in mammals (Barad et al., 1998, Lugnier, 2006). However, the exact mechanism of how PDEs regulate the formation of memory remains to be discovered.

Here, we used the fruit fly *Drosophila melanogaster* to unravel dynamic features of memory formation and identify general concepts of how the brain sorts information and manifests consolidated long-term memory (LTM). The Drosophila brain features a much lower number of neurons than the brains of mammals while maintaining an important complexity and reproducibility of general memory processes. This has allowed the discovery of several fundamental cellular and molecular processes of memory formation due to the highly developed experimental approaches and powerful genetic tools in Drosophila (Heisenberg, 2003, Keene and Waddell, 2007). For instance, using a classical aversive conditioning paradigm, flies can efficiently learn to avoid an odor that was previously paired with electric shocks, although the obtained memory decays quickly when an odor-shock pairing occurs during a single training cycle. Stable protein-synthesis-dependent LTM is exclusively formed when fruit flies are subjected to a specific conditioning pattern, during which the training cycle is experienced repeatedly and spaced by resting intervals (Tully et al., 1994). In Drosophila, the mushroom body (MB) is the olfactory learning and memory center and plays a crucial role for LTM formation (Pascual and Préat, 2001). The MB consists of approximately 2,000 neurons per hemisphere, whose axons first bundle within the peduncle and then form 5 discrete lobular structures that show functional homologies to the striatum and hippocampus in mammals (Pagani et al., 2009, Wolff and Strausfeld, 2015). Molecular mechanisms that integrate the spacing effect in the MB downstream of the associative process have been demonstrated (Pagani et al., 2009), but how is the spacing effect integrated upstream of the memory center? Context evaluation largely involves the dopaminergic system in mammals as well as in Drosophila (Morris et al., 2006, Zhang et al., 2007). Here, the MB is innervated by a complex but specifically compartmentalized network of dopaminergic neurons (DNs) that modulate MB intrinsic activity during associative learning and can, thus, control specific behavioral output (Aso et al., 2010, Aso et al., 2014, Boto et al., 2014). Recently, our team assigned the gating of LTM to specific DNs, including MP1, that are situated upstream of the MB peduncle and exert a significant increase in activity, both during and shortly after the repeated cycles of spaced conditioning (Plaçais et al., 2012). To date, the identity of regulatory input for the dopaminergic system remains largely elusive; consequently, the initial events that integrate the relevance of an association and trigger LTM are unknown.

The *dunce*^*1*^ (*dnc*^*1*^) PDE loss-of-function mutant was the first memory mutant described in Drosophila and was instrumental in the discovery of the role of cAMP/PKA signaling in memory formation (Dudai et al., 1976). Importantly, it restricts the level of cAMP at the presynapse, a function that is widely accepted to cause deficits in short-lived memory performances once deregulated in a *dnc*^*1*^ mutant (Davis and Dauwalder, 1991). This discrepancy with mammalian phenotypes of memory amelioration after PDE inhibition has hindered the use of Drosophila for determining the specific facilitative actions of PDEs on memory.

Strikingly, we found here that the *dnc*^*1*^ mutation increases PKA activation, which triggers downstream plasticity changes and selectively facilitates LTM. Specifically, we characterized a new pair of serotonergic projection neurons (this pair is collectively abbreviated hereinafter as SPN) that are activated by lack of Dnc in Drosophila. The SPN is located in the gnathal ganglia (GNG) and projects to central brain compartments, where it surrounds the MB peduncle. We show that Dnc is normally inhibited in the SPN specifically upon spaced training. The resulting activation of the SPN triggers oscillatory activity in MP1 dopamine neurons, which controls LTM formation downstream in the MB. Therefore, we report that Dnc represents the default inhibition of neuronal activity by restraining the level of PKA activity, which plays a crucial role as a memory checkpoint in the SPN. By integrating molecular, circuit, and behavioral analyses in a simple invertebrate brain, this work provides a comprehensive mechanism of PDE input in the control of LTM formation.

## Results

### Decreased Dnc PDE Activity in the SPN Facilitates LTM Formation

**Figure 1:**
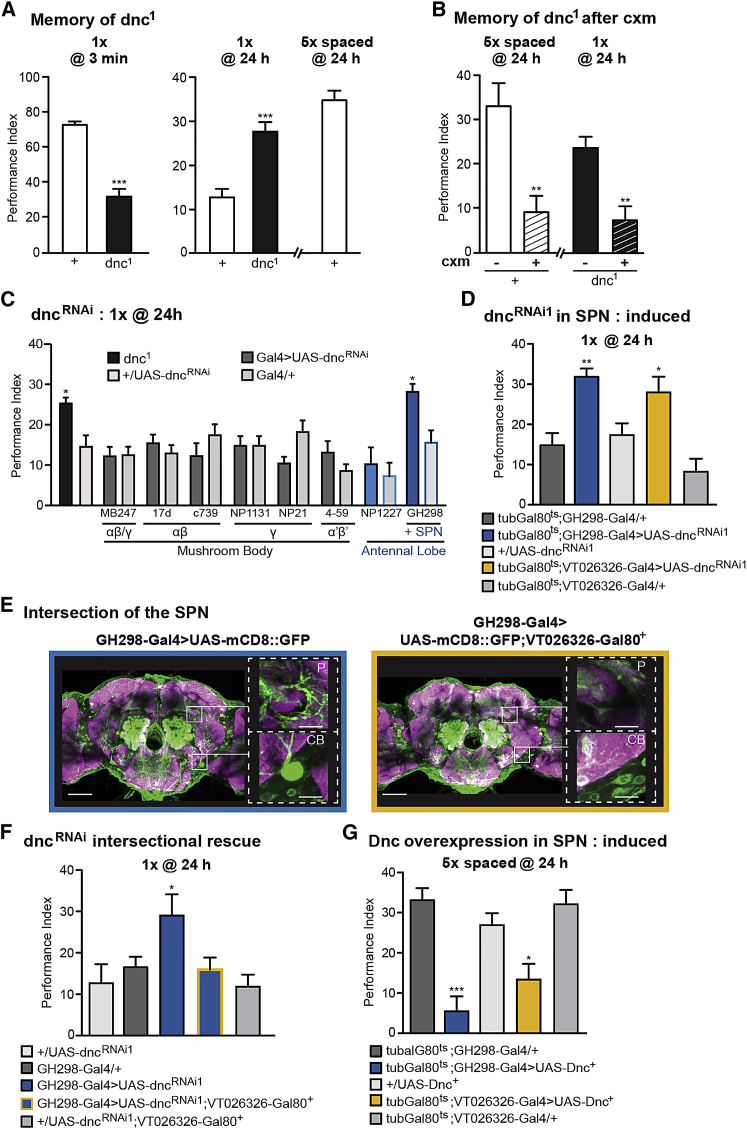
Facilitated Long-Term Memory in Dnc Loss-of-Function Flies (A) After a single training cycle, *dnc*^*1*^ mutants displayed decreased learning scores (t test, t(14) = 8.7, p < 0.0001; n = 8) and increased memory performance at 24 hr in comparison to the Canton-S wild-type control flies (t test, t(17) = 5.1, p < 0.0001; n = 10). The performance level of *dnc*^*1*^ mutant flies after a single training cycle is similar to the LTM score of Canton-S flies after 5′ spaced cycles. (B) LTM of Canton-S flies after 5× spaced cycles was abolished by treatment with the protein synthesis inhibitor cxm (t test, t(14) = 3.75, p = 0.002; n = 8) as well as the high memory performance at 24 hr of *dnc*^*1*^ after a single training (t test, t(14) = 3.77, p = 0.002; n = 8). (C) Memory performance of dnc^RNAi^ in MB α/β and γ neurons, in α/β alone, in γ alone, or in α′/β′ neurons was lower compared to that of *dnc*^*1*^ (F(18, 184) = 5.205, p < 0.0001, n ≥ 8). Dnc^RNAi^ in GH298-Gal4, which labels local interneurons in the antennal lobe (AL) plus a pair of serotonergic projection neurons (SPNs), displayed a significant increase in 24-hr memory performance that was not observed when expression was limited to AL interneurons using NP1227-Gal4. (D) Flies expressing Dnc^RNAi1^ in the SPN using tub-Gal80^ts^;GH298-Gal4 or tub-Gal80^ts^;VT026326-Gal4 displayed increased memory scores at 24 hr after 1 cycle of training (F(4, 64) = 11.53, p < 0.001, n ≥ 9). (E) The SPN is labeled with GFP (green) using GH298-Gal4 (white squares; CB, cell body in the gnathal ganglia; P, projection surrounding MB peduncle), but SPN expression is turned off in the presence of the VT026326-Gal80^+^ transgene. Scale bars, 50 μm (large) and 10 μm (small). (F) GH298>UAS-dnc^RNAi^;VT026326-Gal80^+^ flies did not exhibit any increase in memory performance, contrary to GH298>UAS-dnc^RNAi^ flies (F(4, 45) = 3.39, p = 0.017, n ≥ 9). (G) Overexpression of Dnc in the SPN using tub-Gal80^ts^;GH298-Gal4 and tub-Gal80^ts^;VT026326-Gal4 impairs LTM (F(4, 56) = 8.66, p < 0.001, n ≥ 10). Data are presented as mean ± SEM. ^∗^p < 0.05; ^∗∗∗^p < 0.0001; ns, not significant. Statistical tests in (C), (D), (F), and (G) were performed using one-way ANOVA. Asterisks indicate the least significance level in a Newman-Keuls post-hoc comparison of indicated groups. See also Figure S1, Table S1, and Video S1.

Although *dnc*^*1*^ mutants have impaired learning scores (Dudai et al., 1976) (Figure 1A), they remarkably showed increased memory scores after a single training cycle, as compared to normal flies at 24 hr, the time point generally used to assess LTM (Figure 1A). Our first aim was to confirm that the high level of 24-hr memory observed in *dnc*^*1*^ indeed corresponds to LTM. Cycloheximide (cxm) treatment inhibits the *de novo* protein synthesis underlying LTM (Tully et al., 1994), resulting in its impairment. As expected, cxm treatment precluded the formation of LTM in wild-type flies after 5× spaced training cycles; interestingly, the elevated memory scores in *dnc*^*1*^ were also sensitive to cxm (Figure 1B). This demonstrates that a decrease in Dnc activity allows LTM formation after 1 cycle, whereas LTM is normally formed only after at least 5× spaced cycles.

Next, we aimed to localize this facilitation effect using RNAi to inhibit Dnc expression in specific subsets of brain neurons. Since the MB encodes aversive LTM (Pascual and Préat, 2001, Yu et al., 2006), we chose this structure as our first Dnc knockdown target. The MB neurons can be subdivided based on their axonal morphology into γ, α/β, and α′/β′ subtypes, of which the α/β neurons have repeatedly been shown to specifically support LTM (Pascual and Préat, 2001, Bouzaiane et al., 2015). We addressed the role of MB neurons via MB promoter-driven Gal4 expression of UAS-dnc^RNAi^. Surprisingly, no LTM facilitation could be observed when different subsets of MB neurons were targeted (Figure 1C). We extended our search to the antennal lobe (AL), a second-order olfactory center in the Drosophila brain conveying odor information that was previously suggested to have a role in Dnc-dependent memory formation (Scheunemann et al., 2012). Here, by targeting AL type 1 local interneurons with the widely used driver GH298-Gal4 (Keller and Vosshall, 2003), we were able to recreate the facilitation effect of the *dnc*^*1*^ mutant (Figure 1C). However, Dnc inhibition in the same AL interneurons using a second driver, NP1227-Gal4 (Tanaka et al., 2009), did not increase 24-hr memory (Figure 1C). To solve this discrepancy, we re-examined the expression pattern of GH298-Gal4 driving UAS-mcd8::GFP. This revealed labeling of a prominent neuron (SPN) with a large cell body in the GNG and wide arborizations, one of which sends its projection to the MB to surround the MB peduncle (Lin et al., 2012) (Figure S1A; Video S1.1). Based on its proximity to the Drosophila memory center, we suspected that the SPN might be functionally linked to LTM. We identified a second Gal4 line from the Vienna Drosophila Resource Center (VDRC) collection (VT026326) that targets the SPN but none of the AL interneurons (Figure S1A; Video S1.2). We used the SPN Gal4 drivers GH298 and VT026326 and the thermo-inducible Gal80^ts^ expressed under the tubulin promoter, which prevents Gal4 activity at low temperature during development (McGuire et al., 2003), to express UAS-dnc^RNAi^ in the SPN of adult flies. Inhibition of Dnc in the SPN facilitated LTM formation after a single-cycle training (Figure 1D). This suggests that the single paired SPN, and not the AL interneurons, was responsible for the LTM facilitation effect observed in GH298>UAS-dnc^RNAi^ flies (Figure 1C). The facilitation was also present when a second Dnc-targeting RNAi was expressed in the SPN of adult flies (Figure S1B). Furthermore, the memory performance of non-induced flies was similar to that of the genotypic controls (Figure S1C). Thus, Dnc inhibition is required in the adult SPN for LTM formation.

In order to further confirm the neuronal specificity of facilitation in the SPN, we performed a genetic intersection. SPN expression was excluded from the GH298 expression pattern by cloning the promoter of the VT026326 driver in front of the sequence encoding the Gal80^+^ protein that constitutively inhibits Gal4 expression. No SPN labeling was observed in flies that combined VT026326-Gal80^+^ and GH298-Gal4 to drive UAS-mcd8::GFP, whereas the remaining GH298-Gal4 expression pattern was unaffected to the best of our assessment (Figure 1E; Video S1.3). In addition, the facilitation effect of the Dnc knockdown was abolished when Gal4 expression was turned off in the SPN using VT026326-Gal80^+^ (Figure 1F). This intersectional rescue strongly supports the idea that the function of Dnc during LTM formation is executed in the SPN.

We next asked whether the level of active Dnc in the SPN could have either a permissive or a restrictive effect on LTM, and therefore, we overexpressed Dnc in the SPN of adult flies. The results indicate that expression of UAS-Dnc+ in the SPN inhibited LTM after a 5× spaced training protocol (Figure 1G). The non-induced control flies performed normally (Figure S1D). Flies with constitutive Dnc overexpression showed the same LTM defect (Figure S1E). However, flies that submitted to a 5× massed training protocol that only yields long-term anesthesia-resistant memory (LT-ARM), which is a consolidated memory phase that is less stable than LTM (Bouzaiane et al., 2015, Tully et al., 1994), exhibited normal memory scores (Figure S1F). Overexpression of Dnc did not impair naive sensory responses to the training stimuli (Table S1). This indicates that Dnc overexpression in the SPN specifically inhibits LTM, likely via decreased cAMP/PKA signaling in the SPN, whereas decreased Dnc PDE activity facilitates LTM formation. Combined, these results identify Dnc as a major regulator of LTM formation in the SPN.

### The SPN Sends Presynaptic Terminals to the MB Peduncle and Is Involved in LTM Consolidation

**Figure 2:**
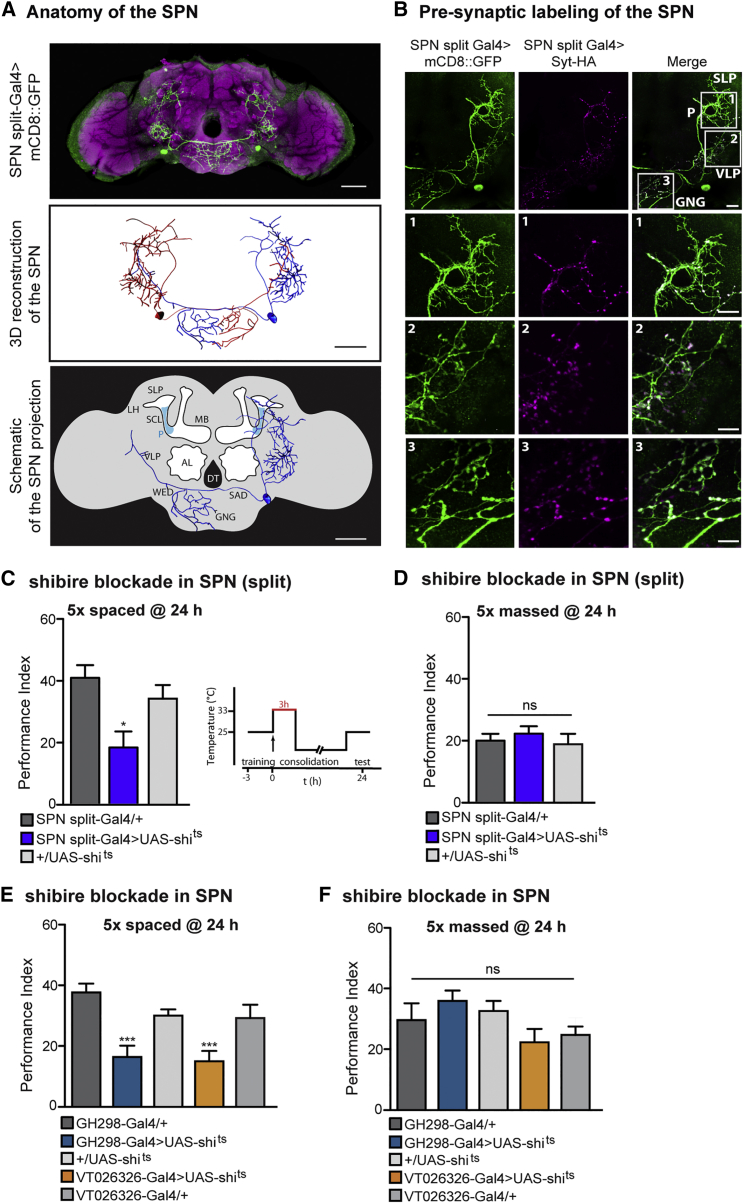
Projection Profile of the SPN and Implication in LTM Consolidation (A) Top: illustration of a fly brain in which the SPN is visualized with SPNsplit-Gal4>UASmCD8::GFP (green) and pan-neuronal anti-nc82 counterstaining (magenta). The SPN cell body is visible in the gnathal ganglia (GNG). Projections include a loop that surrounds the MB peduncle. Scale bar, 50 μm. Middle: 3D reconstruction using Imaris to visualize individual SPN projection from the left brain hemisphere (red) and right brain hemisphere (blue). Scale bar, 50 μm. Bottom: the SPN has contralateral projections near the digestive tube (DT) into the GNG and saddle (SAD). The SPN projects ipsilaterally into the ventral-lateral protocerebrum (VLP), the superior clamp surrounding the MB peduncle (SCL), the superior lateral protocerebrum (SLP), and lateral horn (LH). P, MB peduncle. Scale bar, 50 μm. Descriptions of brain regions also correspond to the contralateral site. (B) SPN polarity was visualized driving mCD8::GFP (green) and Syt::HA (magenta), a specific presynaptic marker, under the control of SPNsplit-Gal4. Presynaptic terminals localize to processes in the GNG, the VLP, SCL (MB peduncle), and SPL. (C) Memory performance was impaired in SPNsplit-Gal4 > UAS-shi^ts^ flies (F(2, 27) = 7.41, p = 0.003; n ≥ 9) after 5× spaced training, when flies were subjected to elevated temperature for 3 hr immediately after the last training cycle (see detailed scheme with temperature regime). (D) After massed training, no difference in memory scores was observed (F(2, 27) = 0.61, p = 0.55; n ≥ 9). (E) Memory performance was impaired after 5× spaced training in VT026326>UAS-shi^ts^ and GH298>UAS-shi^ts^ (F(4, 57) = 8.9, p < 0.0001; n ≥ 10) when flies were subjected to the same temperature regime as described in (C). (F) After massed training, no difference in memory scores was observed (F(4, 44) = 2.4, p = 0.062; n ≥ 8). Data are presented as mean ± SEM. ^∗^p < 0.05; ^∗∗^p < 0.01; ^∗∗∗^p < 0.0001; ns, not significant. Statistical tests were performed using one-way ANOVA. Asterisks indicate the least significance level in a Newman-Keuls post-hoc comparison of indicated groups. See also Figure S2 and Videos S2 and S3.

To examine the individual properties of the SPN, we generated a split-Gal4 line that expresses uniquely within the SPN (Figure 2A; Video S2). Using Imaris 3D reconstruction, we individually traced the projections of the two bilateral neurons for a more thorough characterization (Figure 2A; Video S3). We determined SPN projection sites in defined neuropil regions of the Drosophila brain according to the classification by the Insect Brain Name Working Group (Ito et al., 2014). Briefly, from the cell body located in the lateral part of the GNG (at its border) to the wedge (WED) and the saddle (SAD), we observed a prominent ipsilateral projection surrounding the MB peduncle, where it forms a circle at the superior clamp (SCL). Furthermore, the SPN projects contralaterally into the GNG and the SAD, crossing the brain close to the digestive tube (DT). The SPN also displays strong ipsilateral arborization in the anterior and posterior ventrolateral protocerebrum (VLP) and the superior lateral protocerebrum (SLP), from where it contacts the lateral horn (LH). Subsequently, we sought to identify presynaptic terminals of the SPN in order to determine where it might perform its specific function during LTM. Staining of the SPN using the SPNsplit-Gal4>UASmcd8::GFP along with the pre-synaptic marker synaptotagmin (Zhang et al., 2002) revealed presynaptic sites that are located (1) at the superior clamp surrounding the MB peduncle (P in Figure 2B) and the SLP, (2) in the VLP, and (3) throughout the GNG and SAD (Figure 2B).

To determine whether SPN neurotransmission is directly implicated in LTM formation, we expressed the widely used thermo-sensitive dynamin allele shibire^ts^ (shi^ts^), which prevents synaptic transmission (Kitamoto, 2002), in the SPN using the split-GAL4 line. We found that flies trained for LTM, but in which SPN transmission was blocked for 3 hr immediately after the last cycle of the 5× spaced training protocol, had a strong 24-hr memory deficit (Figure 2C). Furthermore, the same blocking protocol after 5× massed conditioning did not affect LT-ARM scores (Figure 2D). We found the same memory defect for LTM using the two SPN Gal4 lines GH298 and VT026326 (Figure 2E), whereas LT-ARM performance was normal (Figure 2F). In addition, control LTM experiments conducted at the permissive temperature did not reveal any memory impairment (Figures S2A and S2B). This indicates that blocking the SPN during the consolidation phase impairs LTM but not LT-ARM. An intersectional rescue experiment using VT026326-Gal80^+^ was able to restore LTM memory capacities by turning off shi^ts^ expression in the SPN (Figure S2C). These results reveal a role for neurotransmission from the SPN in LTM within the initial period of memory consolidation.

### The SPN Is Serotonergic

**Figure 3:**
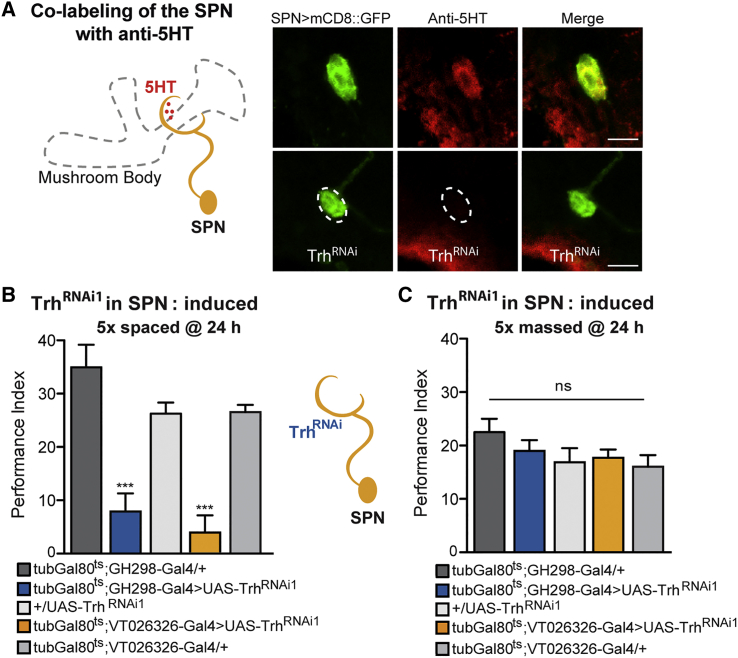
LTM Relies on 5HT Signaling from the 5HT-Positive SPN (A) Left: Scheme of the SPN and the circular projection surrounding the mushroom body peduncle. Right: The cell body of the SPN visualized using UAS-mCD8::GFP (green) co-localizes with a marker for serotonin (anti-5HT, red). Knockdown of tryptophan-hydroxylase (Trh) using Trh^RNAi1^ reduced the 5HT signals in the SPN cell body. Scale bars, 10 μm. (B) Trh knockdown in SPN in adult flies impaired LTM, using either tub-G80^ts^;GH298-Gal4 (F(2, 26) = 16.61, p < 0.0001; n ≥ 8) or tub-G80^ts^;VT026326-Gal (F(2, 31) = 32.45, p < 0.0001; n ≥ 8). (C) Memory performance at 24 hr after massed training was not impaired (F(4, 42) = 1.37, p = 0.26; n ≥ 8). Data are presented as mean ± SEM. ^∗∗∗^p < 0.0001; ns, not significant. Statistical tests were performed using one-way ANOVA. Asterisks indicate the least significance level in a Newman-Keuls post-hoc comparison of indicated groups. See also Figure S3 and Table S1.

The previous finding prompted us to identify the transmitter released by the SPN. We detected a strong overlap of serotonin antibody (anti-5HT) and GFP labeling of the SPN at the level of the SPN cell body (Figure 3A). Consistently, the anti-5HT signal was strongly reduced by knockdown of the 5HT synthesis-limiting enzyme tryptophan-hydroxylase (Trh) in the SPN via Trh^RNAi1^ (Figure 3A).

To address the function of 5HT release by the SPN and its involvement in LTM formation, we expressed the UAS-Trh^RNAi1^ construct under the control of thermo-inducible versions of the two SPN Gal4 drivers, GH298 and VT026326. Reduced 5HT signaling in the adult SPN strongly impaired LTM after 5× spaced training (Figure 3B) but not LT-ARM after 5x massed training (Figure 3C). The same result was obtained with a second non-overlapping RNAi against Trh (Figures S3A and S3B). Non-induced flies did not display any LTM memory deficit (Figure S3C), and the Trh knockdown did not impair odor acuity or shock response of the flies (Table S1). These results indicate that serotonin expression is required specifically in the SPN for normal LTM.

### The SPN Connects MP1 Dopaminergic Neurons at the Level of the MB Peduncle

**Figure 4:**
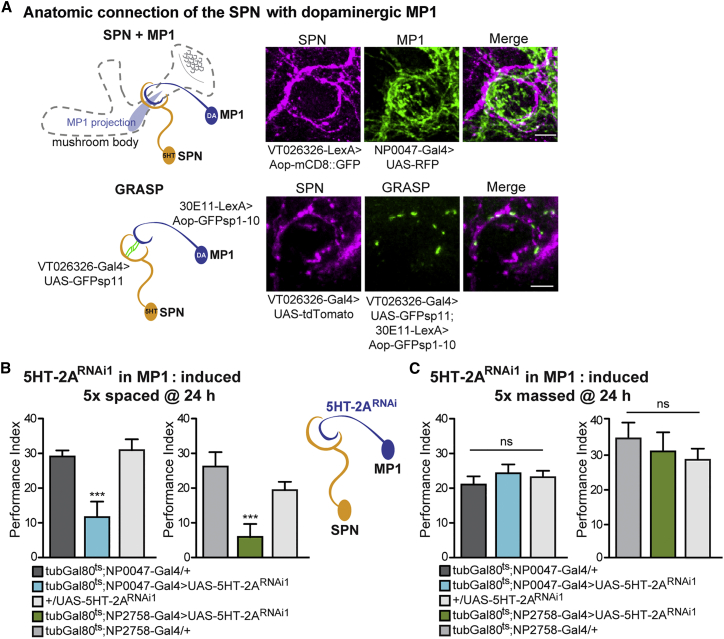
Anatomical and Behavioral Analysis of the SPN Connection to the Dopaminergic MP1 (A) Left: Scheme of the SPN and MP1 and their projection sites at the mushroom body peduncle. Right: SPN (green) and MP1 (magenta) were simultaneously visualized using VT026326-LexA>Aop-mCD8::GFP and NP0047-Gal4>UAS-RFP, respectively. GFP Reconstitution Across Synaptic Partners (GRASP) revealed reconstituted GFP signals (green) at the level of the SPN projection around the MB peduncle (magenta). Scale bars, 5 μm. (B) Induced knockdown of the serotonergic receptor 5HT-2A in MP1 impaired LTM, using either tub-G80^ts^;NP0047 (F(2, 29) = 10.51, p = 0.0005; n ≥ 9) or tub-G80^ts^;NP2758 (F(2, 28) = 6.9, p = 0.003; n ≥ 9). (C) Memory performance at 24 hr after massed training was not affected in tub-G80^ts^;NP0047>5HT-2A^RNAi1^ (F(4, 50) = 1.08, p = 0.37; n ≥ 8) or in tub-G80^ts^;NP2758 (F(2, 25) = 0.65, p = 0.53; n ≥ 8) flies. Data are presented as mean ± SEM. ^∗∗∗^p < 0.0001; ns, not significant. Statistical tests in (B) and (C) were performed using one-way ANOVA. Asterisks indicate the least significance level in a Newman-Keuls post-hoc comparison of indicated groups. See also Figure S4, Table S1, and Videos S4, S5, and S6.

In a previous report, we were able to assign an LTM-gating mechanism to the dopaminergic MP1 neurons, which project onto the MB peduncle. This occurs through a characteristic pattern of slow calcium oscillation in MP1 neurons, which are prominent after 5× spaced conditioning but not after massed conditioning (Plaçais et al., 2012). The MP1 neuron projects specifically onto MB axons deep in the peduncle that correspond to α/β neurons, as well as onto the heel of γ neurons (Aso et al., 2010, Tanaka et al., 2008). Our results for the SPN prompted us to investigate a possible connection of the SPN and MP1 at the level of the MB peduncle. To address this question, we first conducted a co-labeling experiment of both neurons by taking advantage of the two independent binary targeting systems in Drosophila, Gal4/UAS, and LexA/Aop (Yagi et al., 2010) to simultaneously express two different genetic reporters. To achieve this, we generated a VT026326-LexA line by cloning the VT026326 promoter in front of the LexA sequence. We used this line to express mCD8::GFP in the SPN, while we expressed UAS-RFP using NP0047-Gal4, a driver that has been used in multiple studies to target MP1 neurons (Tanaka et al., 2008). This enabled us to observe the close proximity of the circular projection of the SPN and MP1 processes at the level of the MB peduncle (Figure 4A; Video S4). We then repeated this experiment using another combination of drivers—namely, 30E11-LexA (Plaçais et al., 2017) to express mCD8::GFP in MP1 and VT026326-Gal4 to drive UAS-tdTomato in the SPN. Once again, we observed overlapping labeling of the SPN and MP1 at the level of the MB peduncle (Figure S4A; Video S5).

Next, we performed GFP Reconstitution Across Synaptic Partners (GRASP) (Feinberg et al., 2008) to look for potential synaptic connections between the SPN and MP1 (Figure 4A). For this, two split-GFP constructs were expressed under the control of VT026326-Gal4 (SPN)>GFP_sp11_ and 30E11-LexA (MP1)>GFP_sp1–10_, respectively. Additionally, we visualized the SPN by driving the genetic reporter tdTomato. Using these approaches, we detected GRASP signals localized to the SPN projection at the MB peduncle (green) (Figure 4A; Figure S4A; Video S6). These experiments suggest that SPN and MP1 contact each other at the level of the MB peduncle.

We reasoned that the SPN might control the LTM gating process by directly controlling MP1 activity. Accordingly, we looked for serotonin receptor expression in MP1 that could be involved specifically in LTM formation. Knockdown of the excitatory 5HT-2A receptor (Sheldon and Aghajanian, 1991) in MP1 neurons of adult flies using either the NP0047 or NP2758 driver resulted in an LTM defect after 5× spaced conditioning (Figure 4B), whereas LT-ARM remained normal after massed training (Figure 4C). The same result was achieved using a second non-overlapping RNAi to knock down 5HT-2A in MP1 (Figures S4B and S4C). Non-induced control flies did not show any memory deficit, and sensory tests were normal for all genotypes (Figure S4D; Table S1). These results indicate that serotonergic signaling onto MP1 neurons is involved in LTM formation. The dorsal paired medial (DPM) neuron is another prominent serotonergic neuron connected to the MB that is known to be involved in associative olfactory memory (Lee et al., 2011), which could make contact with the MP1 neurons. However, blocking DPM synaptic transmission directly after LTM training, during the period when MP1 neurons are required for LTM (Plaçais et al., 2017), did not affect LTM performance (Figure S4E). This further prompted us to presume that the SPN sends its serotonergic projections toward the MB peduncle, where they can signal onto the dopaminergic MP1 via the 5HT-2A receptor.

Previous reports have shown that other types of dopaminergic neurons that project onto the MB lobes can regulate locomotor activity in Drosophila (Berry et al., 2015, Cohn et al., 2015). Therefore, we examined whether the SPN-MP1 neuron circuit studied here could have a similar role. We disrupted this neuronal axis using three distinct manipulations (5HT-2A knockdown in MP1, blockade of the SPN using sibire^ts^, and Dnc knockdown in SPN neurons), none of which could alter the locomotor activity (Figures S4F–S4H).

Thus, all of the behavioral data presented so far point to a dedicated role for serotonergic signaling from the SPN onto the MP1 neurons via the 5HT-2A receptor in LTM formation, in a 3-hr time window following spaced conditioning. To further challenge the functional connection between the SPN and MP1 neurons, we subsequently investigated the effect that disrupting SPN signaling has on MP1 activity.

### Serotonin from the SPN Controls the Frequency of MP1 Oscillations

**Figure 5:**
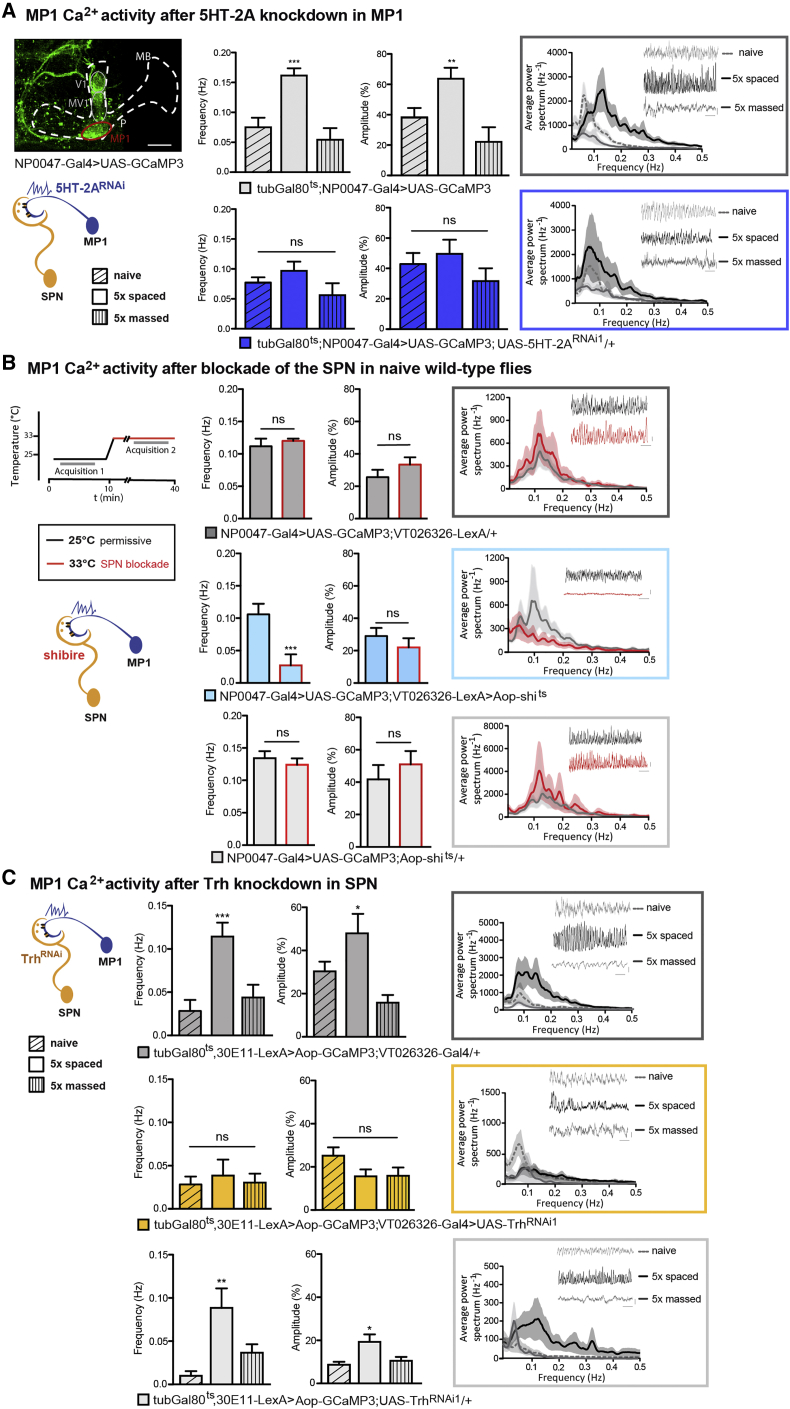
MP1 Ca^2+^ Oscillations Are Sensitive to 5HT Signals from the SPN (A) Ca^2+^ activity in MP1 was observed with UAS-GCaMP3 driven by tub-Gal80^ts^;NP0047-Gal4 (imaging plane in red circle). Control flies displayed the characteristic enhanced oscillatory activity pattern after 5× spaced training, in comparison to naive flies or flies receiving 5× massed training (frequency: F(2, 22) = 17.65, p < 0.0001; amplitude: F(2, 22) = 11.46, p = 0.0006; n = 6–8). Flies expressing 5HT-2A^RNAi1^ in MP1 at adulthood displayed no significant differences in MP1 Ca^2+^ activity between naive, 5× spaced, or 5× massed training conditions (frequency: F(2, 22) = 1.85, p = 0.18; amplitude: F(2, 22) = 1.25, p = 0.39; n = 6–8). Power spectra are shown for the three conditions, along with representative actual time traces for each condition (horizontal bar: 50-s timescale, vertical bar: 20% relative fluorescence change). (B) When synaptic transmission from the SPN was blocked using Aop-shi^ts^, the MP1 Ca^2+^ oscillation frequency decreased (frequency: t test, t(14) = 3.3, p = 0.005; n = 9; amplitude: t test, t(14) = 0.03, p = 0.9; n = 9). In control flies, no significant effect of the elevated temperature regime was observed for VT026326-LexA/+ (frequency: t test, t(12) = 0.75, p = 0.46; amplitude: t test, t(12) = 1.1, p = 0.28; n = 8) and UAS-shi^ts^/+ (frequency: t test, t(12) = 0.69, p = 0.5; amplitude: t test, t(12) = 0.76, p = 0.46; n = 8). Power spectra and time traces are shown as described in (A). (C) Flies with induced Trh knockdown in SPN to decrease serotonin synthesis failed to increase Ca^+^ activity in MP1 after 5× spaced training compared to naive or 5× massed training (frequency: F(2, 21) = 0.26, p = 0.77; amplitude: F(2, 21) = 2.56, p = 0.11; n = 6–8). In control flies, a significant increase in Ca^2+^ activity after spaced training was observed for VT026326-Gal4/+ (frequency: F(2, 21) = 4.92, p = 0.02; amplitude: F(2, 21) = 10.94, p = 0.001; n = 6–8) and UAS-Trh^RNAi1^/+ (frequency: F(2, 21) = 8.93, p = 0.002; amplitude: F(2, 21) = 4.74, p = 0.002; n = 6–8). Power spectra and time traces are shown as described in (A). Data are presented as mean ± SEM. ^∗^p < 0.05; ^∗∗^p < 0.01; ^∗∗∗^p < 0.0001; ns, not significant. Statistical tests in (A) and (C) were performed using one-way ANOVA. Asterisks indicate the least significance level in a Newman-Keuls post-hoc comparison of indicated groups. See also Figure S5.

We asked whether serotonergic signaling could be responsible for the LTM-dependent switch in oscillation frequency in MP1 neurons. To address this, we performed a 5HT-2A-receptor knockdown in adult flies as in the previous behavior experiments, which additionally co-expressed GCaMP3 in MP1 neurons to visualize Ca^2+^ activity for *in vivo* imaging (Figure 5A). Control flies expressing the Ca^2+^ sensor without 5HT-2A^RNAi^ displayed the expected increase in frequency and amplitude of MP1 calcium oscillations after 5× spaced training as compared to naive flies, and massed training decreased the amplitude of MP1 neuronal activity (Plaçais et al., 2012) (Figure 5A). By contrast, under 5HT-2A knockdown conditions, the pattern of spontaneous calcium activity was similar in flies after spaced conditioning and in naive flies, although massed training still resulted in decreased activity (Figure 5A). Using a second RNAi construct (5HT-2A^RNAi2^), we confirmed that the oscillatory activity in MP1 neurons after spaced training was markedly reduced (Figure S5A). We observed relatively large variations of calcium traces in naive flies. To ensure that the effect of increased frequency and amplitude of MP1 oscillations after spaced training was specific, we performed an inner-group cross-validation of naive flies and observed no significant differences in MP1 activity (Figure S5B). Taken together, our data reveal a functional connection between MP1 and the serotonergic SPN via the 5HT-2A receptor, which is critically involved in regulating the central LTM gating mechanism of MP1.

To further address the SPN-MP1 functional link, we examined whether blocking SPN synaptic transmission was sufficient to alter MP1’s steady-state activity. For this, we simultaneously expressed shi^ts^ in the SPN and the GCaMP3 Ca^2+^ sensor in MP1. We recorded Ca^2+^ activity before and after onset of the restrictive temperature in single flies and detected a strong decrease in the frequency of MP1 Ca^2+^ activity due to the blockade of SPN transmission (Figure 5B). These results indicate that blocking synaptic transmission from the SPN can alter MP1 spontaneous activity in naive flies. In order to exclude whether blockade of the serotonergic DPM neuron that innervates the MB could similarly affect MP1 activity, we conducted the same experiment expressing shi^ts^ in the DPM. After DPM blockade, no changes in the frequency or amplitude of MP1 calcium oscillations were detected (Figure S5C).

Since reduced serotonin signaling in the SPN impaired LTM formation (Figure 3B), our model predicts that Trh knockdown in the SPN should block the MP1 gating mechanism by abolishing the increase in oscillation after spaced conditioning. Therefore, we expressed Trh^RNAi1^ in the SPN of adult flies simultaneously with GCaMP3 to conduct *in vivo* Ca^2+^ imaging of MP1. These flies failed to show any increase in Ca^2+^ oscillation frequency or amplitude normally displayed by MP1 in response to 5× spaced training cycles, as compared to massed training or to naive flies (Figure 5C). A second RNAi confirmed that Trh knockdown in the SPN prevented the enhancement of oscillatory activity in MP1 neurons after spaced training (Figure S5D). Therefore, blocking SPN transmission through shi^ts^ expression, as well as interfering with serotonin signaling in the SPN, modulates MP1 activity and abolishes the LTM gating function of MP1.

### SPN Activation Enhances the Frequency of MP1 Oscillations and Facilitates LTM

**Figure 6:**
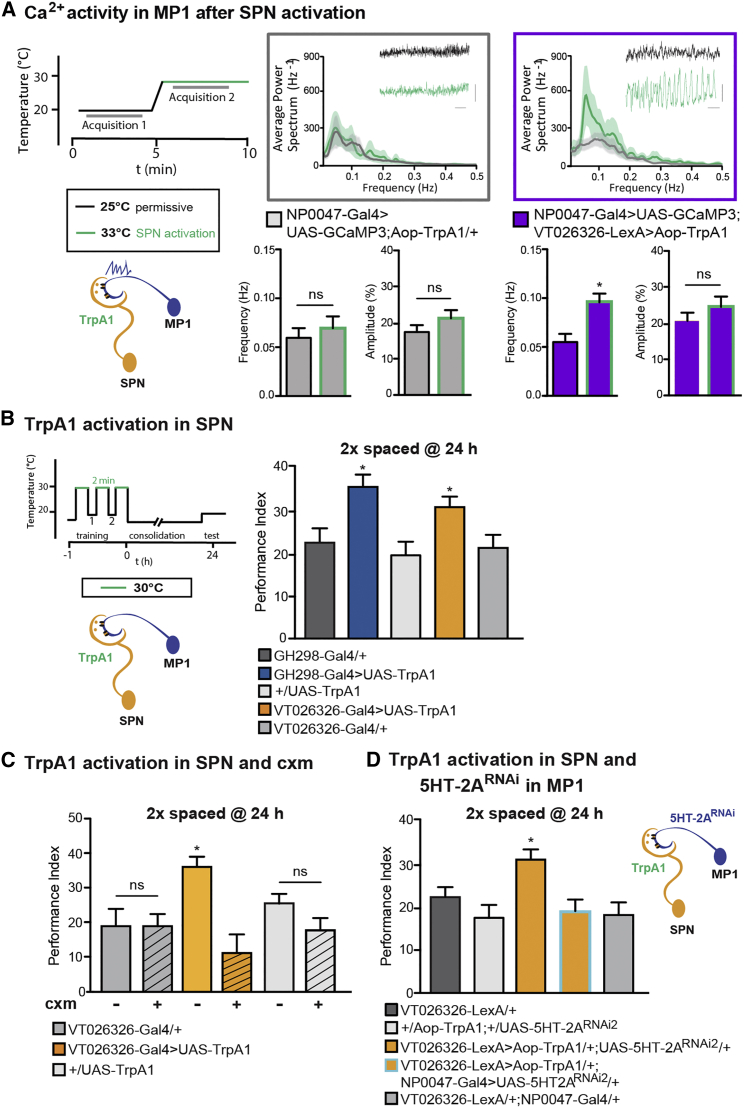
Activation of the SPN Enhances MP1 Oscillation and Facilitates LTM (A) Ca^2+^ activity was observed in MP1 expressing GCaMP3 with NP0047-Gal4. The SPN was activated using the thermo-sensitive cation channel dTrpA1. dTrpA1 activation of the SPN elicited a significant increase in Ca^2+^ activity of MP1 oscillations, t(9) = 2.7, p = 0.024, paired t test; n = 10. Control flies did not exhibit any significant effect in Ca^2+^ activity in response to the elevated temperature, t(10) = 0.85, p = 0.41, paired t test; n = 10. (B) Flies were subjected to a 2× spaced-cycle conditioning interspersed with periods of thermal activation as illustrated. The 24-hr memory performance of VT026326>UAS-dTrpA1 and GH298-Gal4>UAS-TrpA1 flies was increased as compared to the genotypic controls (F(5, 55) = 4.43, p < 0.002, n ≥ 9). (C) The increase in memory performance in activated VT026326>UAS-dTrpA1 flies was abolished by cxm treatment (F(5, 60) = 4.1, p < 0.003; n ≥ 10), whereas the control flies showed no differences in memory performance between the cxm-treated and untreated conditions. (D) The increase in memory performance in activated VT026326>UAS-dTrpA1 after 2× cycles was abolished by expression of 5HT-2A RNAi in MP1 (F(4, 59) = 4.7, p < 0.009; n ≥ 11). Data are presented as mean ± SEM. ^∗^p < 0.05; ns = not significant. Statistical tests in (B), (C), and (D) were performed using one-way ANOVA. Asterisks indicate the least significance level in a Newman-Keuls post-hoc comparison of indicated groups. See also Figure S6.

Blocking transmission from the SPN interfered with LTM formation and disabled MP1 activity (Figures 2C and 5B), which is in line with our other observation that the signal is transduced by the excitatory 5HT-2A receptor (Figures 4B and 5A). To determine whether activation of the SPN is sufficient to enable MP1 oscillation and generate LTM, we expressed dTrpA1, a thermo-sensitive cation channel that allows stimulation of neurons at a specific temperature window (Hamada et al., 2008) in the SPN, and we simultaneously expressed GCaMP3 to visualize Ca^2+^ activity in MP1. SPN activation successfully increased the frequency of naive spontaneous Ca^2+^ activity in MP1, whereas this increase was not apparent in control flies (Figure 6A). This suggests that the accelerated phasic activity in MP1 is due to activation of the SPN and not a general increase in activity as a result of the temperature shift.

We then asked whether activation of the SPN was sufficient to facilitate LTM by controlling the LTM gating function of MP1. We hypothesized that activating the SPN before, during, and after a 2× spaced training protocol would mimic the 5× spaced training cycles of the LTM training protocol. Indeed, flies that were subjected to this training protocol showed increased memory at 24 hr (Figure 6B), whereas trained control groups held at low temperature did not show any difference in memory performances (Figure S6A). To ensure that the increased memory corresponds to protein-synthesis-dependent LTM, we conducted cxm treatment, which, in fact, occluded the additional memory that is formed after SPN activation (Figure 6C). Moreover, we were able to occlude the increase in memory formation after SPN activation by simultaneous expression of RNAi against the 5HT-2A receptor in MP1 (Figure 6D). Non-induced control flies displayed normal performance after 24 hr (Figure S6B). These results demonstrate that SPN activation is both necessary and sufficient to induce LTM and that it specifically acts via 5HT-2A receptors in MP1.

### SPN Activity Counteracts Anesthesia-Resistant Memory Formation

**Figure 7:**
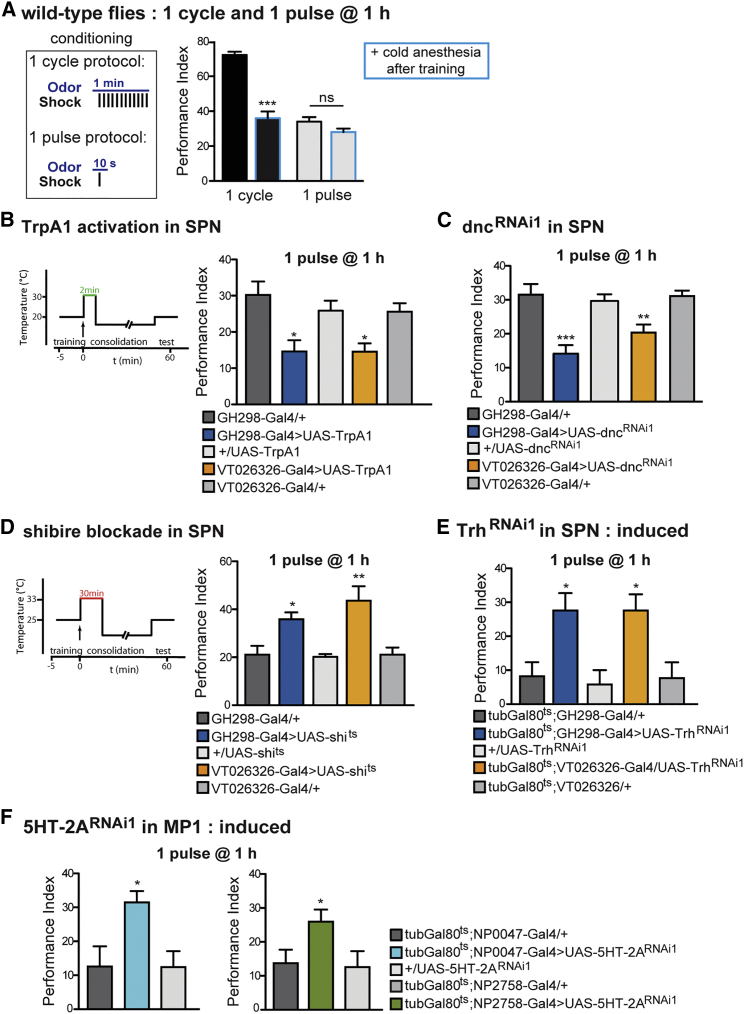
SPN Activity Counteracts ARM Formation (A) Memory performance of Canton-S wild-type flies at 1 hr after 1 cycle of training, or 1-pulse training with and without cold anesthesia treatment. After 1 cycle of training, flies lost about 50% of their performance with anesthetic treatment; the persistent memory corresponds to ARM. After 1-pulse training, flies only built ARM and did not change their performance after the anesthetic treatment (F(3, 42) = 65.67, p < 0.001; n ≥ 9). (B) SPN activation using TrpA1 after 1-pulse training impairs memory performance at 1 hr (F(4, 62) = 6.1, p = 0.0005; n ≥ 9). (C) Flies receiving Dnc knockdown in the SPN perform poorly after 1 pulse at 1 hr (F(4, 48) = 9.9, p < 0.0001; n ≥ 8). (D) In contrast, flies exhibiting SPN blockade after 1-pulse training using UAS-shi^ts^ showed increased memory after 1 hr (F(4, 49) = 7.4, p < 0.0001; n ≥ 9). (E) Similarly, flies with induced expression of Trh RNAi in the SPN displayed increased memory scores at 1 hr (F(4, 45) = 5.9, p = 0.0008, n = 8). (F) Knockdown of the 5HT-2A receptor in MP1 increased memory performances, using either NP0047-Gal4 (F(2, 28) = 5.3, p = 0.01, n ≥ 9) or NP2758-Gal4 (F(2, 27) = 3.5, p = 0.04, n ≥ 9). Data are presented as mean ± SEM. ^∗^p < 0.05; ^∗∗^p < 0.01; ^∗∗∗^p < 0.0001; ns, not significant. Statistical tests were performed using one-way ANOVA. Asterisks indicate the least significance level in a Newman-Keuls post-hoc comparison of indicated groups. See also Figure S7.

Up until this point, we observed memory performances solely at 24 hr. Notably, previous reports have shown that activation of MP1 leads to memory deficits at shorter time points (Berry et al., 2012, Plaçais et al., 2012). To address this apparent contradiction, we examined whether SPN activation could fit with our previous findings demonstrating that MP1 activation promotes LTM at 24 hr (Plaçais et al., 2017), while it is deleterious for anesthesia-resistant memory (ARM) at shorter time points (Plaçais et al., 2012). ARM is a form of memory present directly after training that can withstand anesthetic cold treatment (Knapek et al., 2011). We assessed memory performances at 1 hr using a newly established training paradigm in which a single shock pulse is presented during 10 s of odor delivery (Scheunemann et al., 2013). This paradigm produces ARM as a unique memory component. Thus, memory performance after a single pulse does not decrease with anesthetic cold treatment, unlike what is observed after a complete cycle of training that consists of 12 shock pulses and that produces both ARM and labile memory (Figure 7A). Interestingly, ARM was significantly impaired when we activated the SPN after 1-pulse training (Figure 7B), whereas the flies performed normally in permissive conditions (Figure S7A). The same effect was observed for Dnc knockdown in the SPN, a genetic manipulation supposedly leading to activation of the cAMP pathway as well as the SPN (Figure 7C). Conversely, blocking transmission from the SPN using shi^ts^ after a 1-pulse training boosted ARM (Figure 7D), whereas flies under permissive conditions performed normally (Figure S7B). Based on our previous results, we expected to find a similar boost of ARM after Trh knockdown in the SPN as well as 5HT-2A knockdown in MP1, which was, indeed, the case (Figures 7E and 7F). Furthermore, non-induced controls were normal for the two conditions (Figures S7C and S7D). Therefore, these results demonstrate that activating the SPN-MP1 axis provides short-term inhibition of ARM, whereas it facilitates LTM. Conversely, blocking the SPN-MP1 axis boosts ARM and impairs LTM.

### Dnc PDE Inhibition in the SPN Occurs Specifically after Spaced Training

**Figure 8:**
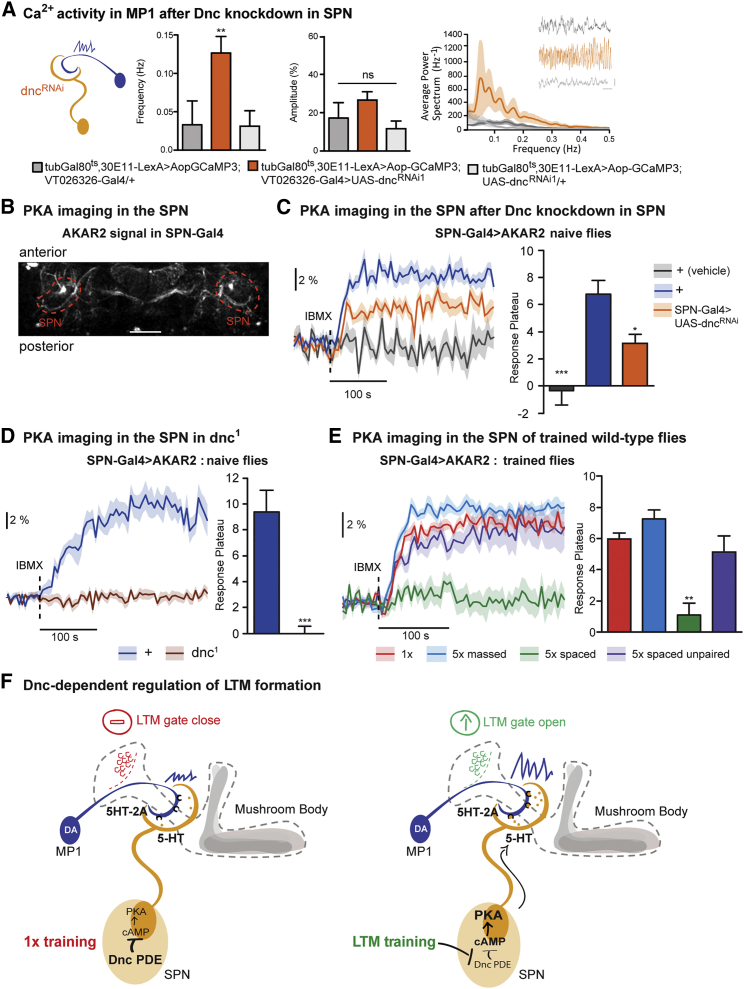
Dnc PDE Inhibition in the SPN Occurs Specifically after Spaced Training (A) Naive flies with induced Dnc knockdown in the SPN showed a significant increase in calcium oscillatory activity in MP1, as compared to the genetic controls (F(2, 24) = 11.18, p < 0.006; n ≥ 8). (B) *In vivo* PKA imaging was conducted on flies expressing UAS-AKAR2 in SPN using VT057280 (red circles). (C) Time traces of PKA activity are shown upon IBMX application on the brain (dashed line) to inhibit PDE. The vehicle solution alone without IBMX did not produce any variation in the AKAR2 signal (n = 5). In response to IBMX, wild-type flies displayed a significant increase in PKA activity (n = 9) that was impaired by the expression of dnc^RNAi^ in the SPN (F(2, 20) = 13.83, p = 0.0002; n = 7). (D) The PKA activation following IBMX application was also observed in wild-type males and was completely abolished in *dnc*^*1*^ hemizygous males (t test, t(11) = 5.589, p = 0.0002; n = 7). (E) IBMX application failed to elicit an increase in PKA activity after 5× spaced training, but not after 1 cycle of training, 5× massed training, or unpaired spaced training (F(3, 38) = 12.77, p < 0.0001; n ≥ 8). (F) Model of the SPN-MP1-MB axis that controls the formation of LTM. Data are presented as mean ± SEM. ^∗^p < 0.05; ^∗∗^p < 0.01; ^∗∗∗^p < 0.0001; ns, not significant. The statistical tests in (A) and (D) were performed using a t test. Statistical tests in (C) and (E) were performed using one-way ANOVA, and asterisks indicate the least significance level in a Newman-Keuls post-hoc comparison of indicated groups. See also Figure S8.

Initially, we demonstrated that decreased Dnc activity in the SPN was sufficient to facilitate LTM in a single-cycle training that yields only short-lived memories in wild-type flies. Having established a functional connection between the SPN and MP1 neurons, we asked whether Dnc knockdown in the SPN could be sufficient to activate MP1 in naive flies. We simultaneously expressed dnc^RNAi^ in the SPN and GCaMP3 in MP1 to image Ca^2+^ activity and observed enhanced MP1 Ca^2+^ oscillations as compared to control flies (Figure 8A). The same increase in MP1 oscillations was observed in naive *dnc*^*1*^ mutant flies (Figure S8A). Therefore, Dnc inhibition in the SPN alone enables sustained MP1 oscillations in naive flies and thereby facilitates LTM formation after a single training cycle.

Thus, we hypothesized that inhibition of Dnc represents an initial step in LTM formation and that, under normal conditions, Dnc inhibition occurs during the 5× spaced training in the SPN. To test this, we took advantage of *in vivo* PKA imaging using the fluorescence resonance energy transfer (FRET) probe AKAR2 in Drosophila by ectopically targeting UAS-AKAR2 to the SPN with a third Gal4 (VT057280) exhibiting strong expression in the SPN. VT057280 labeling was verified by GFP staining, and the LTM defect due to SPN blockade using shi^ts^ was reproducible with this driver (Figures S8B–S8E). Once the conditions for FRET imaging in the SPN were established (Figure 8B), we aimed to characterize Dnc activity in the SPN of naive flies. PKA activity was analyzed in the SPN before and after 3-isobutyl-1-methylxanthine (IBMX) treatment, which inhibits all forms of PDE (Bolger et al., 1993, Gervasi et al., 2010). Since Dnc PDE activity reduces the level of cAMP, we expected to see an increase in the AKAR2 signal after PDE inhibition, as a result of increased PKA activation by cAMP. Indeed, we observed a strong increase in PKA activity after PDE inhibition, revealing that PDEs are constitutively active in the SPN to restrict PKA activity. Interestingly, this increase in PKA activity after IBMX treatment was strongly reduced after a specific Dnc knockdown in the SPN (Figure 8C) and was completely abolished in *dnc*^*1*^ mutant flies (Figure 8D). These results demonstrate that a basal activity of PDE exists in the SPN of naive wild-type flies and that the basal cAMP/PKA inhibition is specifically due to Dnc PDE.

Next, we examined whether the basal PKA inhibition by Dnc PDE is released after 5× spaced training in order to allow LTM formation. If Dnc is already inhibited by spaced training, we would expect no further effect from IBMX treatment in flies that underwent a spaced training. Indeed, we observed that IBMX failed to increase PKA activity in the SPN after LTM conditioning (Figure 8E). In contrast, IBMX treatment increased PKA activity in flies that were conditioned with only 1 cycle, conditioned with 5× massed training, or subjected to an unpaired protocol (in which flies receive 5 spaced presentations of odor and shock but not simultaneously) that does not induce associative learning. The lack of response to IBMX following spaced training could also be due to the fact that PKA is already maximally active (or, at least, at a level that saturates the AKAR2 sensor). However, we were still able to observe forskolin-induced activation of PKA in similar flies (Figure S8F), which rules out this latter interpretation. Nevertheless, the forskolin-induced activation was lower than in control flies, which is consistent with the fact that LTM training intrinsically increases the level of PKA activation through Dnc inhibition. Combined, our results establish that active Dnc PDE constantly restrains PKA activity in the SPN of wild-type flies and that spaced training inhibits Dnc activity in the SPN. This leads to the upregulation of PKA activity, which increases the downstream phasic activity of dopaminergic MP1 via 5HT/5HT-2A signaling, thus opening the LTM gate in the MB (Figure 8F).

## Discussion

The brain filters the most important experiences and encodes them into LTM. Deficient filtering underlies many psychological disorders and may lead to indiscriminate remembering or even to the perturbation of the memory process itself. Selective filtering is thought to be executed by the default inhibition of selective neuronal activity (Luczak et al., 2013), although the precise mechanisms remain obscure. We decipher here that the Dnc PDE controls neuronal activity (Tomchik and Davis, 2009, Gervasi et al., 2010, Boto et al., 2014) and represents a limiting step for LTM within a single pair of Drosophila serotonergic neurons.

### Dnc PDE Controls an LTM Checkpoint Acting Upstream of the Olfactory Memory Center

Not only is LTM formation, the ability to evaluate an experience and retain the information over time, involved in forming an individual’s identity over the course of a lifetime, but it is also crucial for the fitness and survival of any organism. However, which mechanisms does the brain utilize to evaluate the relevance of information that will be consolidated into a long-lasting memory? Molecular memory checkpoints, e.g., a default inhibition of neuronal activity that is released only in a relevant context, could effectuate this selected memory consolidation. PDE-4 activity and cAMP degradation have previously been proposed as promising candidates for such a checkpoint (Abel et al., 1998). Nevertheless, it was still necessary to determine *in vivo* that PDE-4-mediated restriction of cAMP and PKA activity are, indeed, released upon the early steps of LTM formation. Thus, several previous studies focused primarily on memory pathways downstream of associative processes (Barad et al., 1998, Malleret et al., 2001). However, to address the issue of context evaluation and modulation of memory storage, it is crucial to identify memory checkpoints that are upstream of brain structures involved in the association between stimuli. We found here that Dnc represents such a memory checkpoint in a serotonergic circuit that controls memory consolidation via modulation of dopaminergic input, upstream of the olfactory memory center in Drosophila.

### The Serotonin-Dopamine Axis in Control of LTM

At the circuit level, we found that Dnc plays a major role as a modulator of network properties by controlling serotonergic release from the SPN, aside from its potential role in memory processes via regulation of cAMP in the MB (Scheunemann et al., 2012). We propose here an integrated mechanism of LTM control in which a salient (alerting) experience leads to inhibition of Dnc in the SPN. The resulting PKA activation leads to serotonin release by SPN terminals, which, in turn, triggers MP1 oscillations and allows LTM formation downstream in the MB (Figure 8).

Notably, the SPN has wide arborizations within the GNG, a region that is relevant for the processing of nutrient stimuli and feeding behavior (Gordon and Scott, 2009). MP1 signaling has been demonstrated to convey energy-related signals that trigger downstream memory processes in the MB for appetitive memories (Krashes et al., 2009, Musso et al., 2015) but, strikingly, also for aversive memories (Plaçais and Preat, 2013, Plaçais et al., 2017). The SPN-MP1 axis, therefore, represents a potential link that connects metabolic state with memory processing.

Is there an equivalent serotonin-dopamine axis involved in aversive LTM in the mammalian brain? While many studies in mammals support the critical role of dopamine signals in reward and positive motivation involving mainly the ventral tegmental area (VTA) and nucleus acumbens (NA) (Berridge and Robinson, 1998), it is increasingly acknowledged that the VTA also transmits signals related to salient but non-rewarding experiences, such as aversive and alerting events (Matsumoto and Hikosaka, 2009, Bromberg-Martin et al., 2010). These dopaminergic pathways—one promoting motivation value and the other encoding alert salience—have been hypothesized to cooperate in order to support adaptive behavior (Bromberg-Martin et al., 2010). Serotonin and dopamine interactions play a key role in neuropsychiatric diseases with symptoms of cognitive decline; and, interestingly, the implication of serotonin in dopamine-dependent cognitive dysfunction has been suggested (Di Giovanni et al., 2010). Dopamine is released after artificial serotonin microinfusion in the VTA; additionally, a 5HT-2A receptor antagonist has been shown to play a role in changes of oscillatory dopamine release by VTA neurons rather than changing baseline dopamine activity (Di Giovanni et al., 2008, Guan and McBride, 1989). Likewise, we demonstrated that knockdown of the 5HT-2A receptor in MP1 abolishes dopamine oscillation but not spontaneous activity. Serotonin is well known to act as a behavioral switch that controls alternative emotional and physiological states across all phyla (Cools et al., 2008, Waggoner et al., 1998). A serotonin-dopamine axis as described here in Drosophila could, therefore, represent a generic design principle that coordinates how metabolic states integrate into behavior control.

### Dnc PDE Represents a Molecular Balance for Memory Dynamics

Historically, the *dnc*^*1*^ mutant has been shown to display a strong memory defect that can be detected immediately after a single training cycle (Dudai et al., 1976); furthermore, this phenotype has been regularly observed (Tully and Quinn, 1985, Scheunemann et al., 2012). Strikingly, our study reveals that the *dnc*^*1*^ mutation, as well as Dnc knockdown by RNAi in the SPN, leads to a facilitation of LTM formation. Initially, it was reported that *dnc*^*1*^ performs poorly in the short term as well as at 24 hr after a single training cycle (Tully and Quinn, 1985). Notably, at the time of the initial report, the conditions had not yet been established to generate protein-synthesis-dependent LTM in wild-type flies, which may explain why the authors did not observe any increased *dnc*^*1*^ performance at 24 hr. However, we cannot exclude the possibility that other factors, such as genetic background effects, could account for these differences in memory scores at 24 hr for the *dnc*^*1*^ mutant used in this study.

According to our findings, Dnc loss of function is not deleterious for memory formation in general. Instead, Dnc-deficient flies exhibit selective facilitation of consolidated LTM. In fact, contradictory results can be found within studies investigating the consequences of reduced PDE activity. In addition to memory deficits, studies on improved memory are found in other insects (Matsumoto et al., 2006) and, remarkably, in mammals (Barad et al., 1998, Ghavami et al., 2006). Thus, several studies have revealed an improvement of memory after PDE-4-specific inhibitor treatment; moreover, PDE inhibitors are known targets for anti-depressive drugs (Ghavami et al., 2006, Halene and Siegel, 2007). Defective Dnc PDE activity may, therefore, link symptoms of psychological disorders with impaired cognitive functions (Elvevåg and Goldberg, 2000). However, the mechanisms involved have remained obscure. Indeed, significant gain in understanding PDE action in memory formation has been hampered by both the complexity of the mammalian brain and the existence of about 100 different types and isoforms of PDEs (Lugnier, 2006).

One open question is how learning and 3-hr memory are impaired in the *dnc*^*1*^ mutant, while LTM is facilitated at 24 hr. Interestingly, we previously showed that Dnc loss of function is specifically linked to defects in ARM forms of Drosophila memory that are measurable immediately after training and at 3 hr (Scheunemann et al., 2012, Bouzaiane et al., 2015). In addition, we demonstrated here that Dnc inhibition in the SPN as well as artificial SPN stimulation impairs ARM. Based on our previous findings, which established that ARM and LTM are exclusive memory phases (Isabel et al., 2004), we hypothesize that ARM and LTM can be oppositely tuned by the activity of Dnc in the SPN-MP1 axis. Nevertheless, we have not yet identified how Dnc could be inhibited in wild-type flies upon intensive LTM training. Interestingly, biochemical data indicate that ERK2 MAP kinases are able to inhibit Dnc activity (MacKenzie et al., 2000). Furthermore, ERK2 mitogen-activated protein (MAP) kinases have been demonstrated to play a crucial role in LTM (Bekinschtein et al., 2010), making them likely candidates for the inhibition of Dnc upon LTM formation.

In conclusion, contrary to most studies that have addressed suppressor mechanisms primarily by pharmacological inhibition that can artificially elevate PKA (Barad et al., 1998, Kuroiwa et al., 2012), we have demonstrated here that inhibition of Dnc in the SPN is a physiological state that gates LTM after intensive training. In addition to the increasing attention given to PDE inhibitors in recent years, due to their memory facilitation role, there is ongoing research on the specific role of PDEs in symptoms of Alzheimer’s disease (Gurney et al., 2015). Our findings presented here, therefore, offer great potential for revealing the complex action of PDEs in the brain.

## STAR★Methods

### Key Resources Table

**Table:** 

REAGENT or RESOURCE	SOURCE	IDENTIFIER
**Antibodies**		

Anti-GFP (rabbit)	Life Technologies	Cat. A11122; RRID: AB_221569
Anti-GFP (“GRASP”, mouse)	Sigma	Cat. G6539; RRID: AB_259941
Anti-nc82 (mouse)	DSHB	Cat. nc82
Anti-5HT (rabbit)	Sigma	Cat. S5545; RRID: AB_477522
Anti-HA (rat)	Roche/Sigma	Cat. 11867423001; RRID: AB_10094468
Anti-dsRed (rabbit)	Clontech	Cat. 632496; RRID: AB_10013483

**Chemicals, Peptides, and Recombinant Proteins**		

4-methylcyclohexanol (98%)	Sigma	Cat. 218405
3-octanol (99%)	Sigma	Cat. 153095
3-isobutyl-1-methylxantine (IBMX)	Sigma	Cat. I5879
Cycloheximide (cxm)	Sigma	Cat. C7698
Forskolin	Sigma	Cat. F6886

**Experimental Models: Organisms/Strains**		

w^+^[1], dnc^1^	Tully and Quinn, 1985	N/A
Dnc RNAi: w[1], P{UAS-dncRNAi}	Aso et al., 2009	N/A
w[1];P{GAL4.MB247}	Aso et al., 2009	N/A
w[1]; P{w[+mW.hs]=GawB}17d	Aso et al., 2009	N/A
y[1] w[67c23]; P{w[+mW.hs]=GawB}Hr39[c739]	Aso et al., 2009	N/A
w[1118]; P{w[+mW.hs]=GawB}fru[NP0021]	Scheunemann et al., 2012	N/A
y[1] w[1]; P{GawB}NP1131 / CyO, P{UAS-lacZ.UW14}UW14	Aso et al., 2009	N/A
w[1]; P{GawB}4-59	Bouzaiane et al. 2015	N/A
w[1]; P{w[+mW.hs]=GawB}GH298	Scheunemann et al., 2012	N/A
y[1] w[1]; P{GawB}NP1227 / CyO, P{UAS-lacZ.UW14}UW14	Scheunemann et al., 2012	N/A
w[1]; P{GawB}NP0047	Plaçais et al. 2012	N/A
w[1]; P{GawB}NP2758	Plaçais et al. 2012	N/A
w[1118]; P{w[+mC]=QUAS-shi.ts1}3	Plaçais et al. 2012	N/A
w[1]; P{y[+t7.7] w[+mC]=UAS-TrpA1(B).K}attP16	Plaçais et al. 2012	N/A
w[1]; P{VT026326-GAL4}attP2	VDRC	201794
w[1]; P{VT57280-GAL4}attP2	VDRC	200916
Dnc RNAi2: y[1] v[1]; P{y[+t7.7] v[+t1.8]=TRiP.GL01226}attP2/TM3, Sb[1]	BDSC	41644
Trh RNAi1: w[1];P{KK101211}VIE-260B	VDRC	105414
Trh RNAi2: y[1] v[1]; P{y[+t7.7] v[+t1.8]=TRiP.JF01863}attP2/TM3, Sb[1]	BDSC	25842
5HT2A RNAi1: w[1];P{KK113173}VIE-260B	VDRC	102105
5HT2A RNAi2: [1] v[1]; P{y[+t7.7] v[+t1.8]=TRiP.JF02157}attP2	BDSC	31882
w1118; P{UASt-AKAR2}	Gervasi et al., 2010	N/A
w[1118]; P{y[+t7.7] w[+mC]=GMR30E11-lexA}attP40	Janelia Farm	N/A
w[1118]; P{y[+t7.7] w[+mC]=L0111-lexA}attP40	Janelia Farm	N/A
w[1118]; P{y[+t7.7] w[+mC]=VT026326-lexA}attP8	This report	N/A
w[1118]; P{BPp65ADZ(VT026326)}attP40; P{BPZpGal4DBDU (VT026326)}attP2	This report	N/A
w[1118]; P{y[+t7.7] w[+mC]=UAS-GCaMP3.T}attP40	Plaçais et al. 2012	N/A
w[1118]; P{y[+t7.7]=JFRC27-13XLexAop2-IVS-GCamp3-p10}su(Hw)}attp5	Pfeiffer et al., 2010	N/A
w[1118]; M{vas-int.Dm}ZH-2A, PBac{y[+]-attP-9A=TrpA1-lexA}VK00005	Pfeiffer et al. 2010	N/A
w[1118]; M{vas-int.Dm}ZH-2A, PBac{y[+]-attP-9A=shi-lexA}VK00005	Pfeiffer et al. 2010	N/A

**Recombinant DNA**		

Vector Gal80: pBPGAL80Uw-6	Pfeiffer et al. 2010	N/A
Vector LexA: pBPnlsLexA::p65Uw	Pfeiffer et al. 2010	N/A
Vector AD (split): apBPp65ADZpUw	Pfeiffer et al. 2010	N/A
Vector BD (split): pBPZpGAL4DBDUw	Pfeiffer et al. 2010	N/A

**Oligonucleotides**		

Primer VT026326 forward: 5′-GGGGACAAGTT TGTACAAAAAAGCAGGCTCGGACCACATTAA AATCAC-3′	This report	N/A
Primer VT026326 reverse: 5′-GGGGACCACTT TGTACAAGAAAGCTGGGTGCTGCCAGATGAT GGCCC-3′	This report	N/A

**Software and Algorithms**		

GraphPad Prism 5	GraphPad Software, San Diego CA, 2007	http://www.graphpad.com/
MATLAB V2013	MathWorks, Natick, MA	https://fr.mathworks.com/
Imaris 8.3.0	Imaris, Bitplane AG, Zurich	http://www.bitplane.com/imaris

### Contact for Reagent and Resource Sharing

Further information and requests for reagents may be directed to, and will be fulfilled by the Lead Contact, Thomas Preat (thomas.preat@espci.fr).

### Experimental Model and Subject Details

*Drosophila melanogaster* wild-type strain Canton Special (CS) and mutant flies were raised on standard medium at 18°C and 60% humidity in a 12 hr light/dark cycle. *Dnc-RNAi* was generated as previously described (Scheunemann et al., 2012). *Dnc-RNAi2*, *Trh-RNAi1+2* and *5HT-2A-RNAi1+2* were obtained from the Vienna Drosophila Resource Center (VDRC; *Trh-RNAi1*, ID 105414 and *5HT-2A-RNAi1*, ID 102105) and from the Bloomington Drosophila Stock Center (Indiana University; dnc^RNAi1^, ID 41644; *Trh*^*RNAi2*^, ID 25842 and *5HT-2A*^*RNAi2*^, ID 31882). Gal4 drivers for expressing the genes of interest included: *MB247*, *c739*, *17d*, *NP1131*, *NP21*, and *4-49* for expression in MB lobes; *NP1227* for expression in AL interneurons; *NP0047* for expression in MP1; and *GH298*, *VT026326* (VDRC, ID 201794) and *VT57280* (VDRC, ID 200916) for expression in SPN*.* LexA drivers used for expressing the genes of interest included *30E11-LexA* for MP1 and *VT026326-LexA* (generated in our laboratory; see protocol below) for the SPN. For behavioral experiments, both males and females were used in mixed groups. For imaging experiments, females only were used. RNAi expression was specifically induced in adults using the TARGET system (McGuire et al., 2003). To achieve RNAi induction, flies were kept at 30°C for 3 days before conditioning, and also up until the memory assay for LTM and LT-ARM analyses. All *dnc*^*RNAi*^ flies and related crosses were kept at 30°C throughout their development (Scheunemann et al., 2012). VT026326-Gal80^+^ was generated in the laboratory in order to specifically block induction in the SPN (see protocol below). The following strains used for the memory experiments were outcrossed to the CS background: *VT026326-Gal4*, *VT057280-Gal4*, *Dnc-RNAi2, Trh-RNAi1+2*, *5HT-2A-RNAi1+2*, *UAS-shibire*^*ts*^ and *UAS-TrpA1*.

### Method Details

#### Behavioral Experiments

Flies were trained using classical olfactory aversive conditioning protocols as previously described (Pascual and Préat, 2001). Training and testing were performed at 25°C and 80% humidity. Conditioning was performed on samples of 25–35 flies aged 3-4 days with 3-octanol (99% purity, Sigma-Aldrich) and 4-methylcyclohexanol (98% purity, Sigma-Aldrich) at 0.360 mM and 0.325 mM, respectively. Odors were diluted in paraffin oil (VWR International). Memory tests were performed using a T-maze apparatus (Tully and Quinn, 1985). Flies were given 1 min to choose between two arms, each delivering a distinct odor. An index was calculated as the difference in the number of flies in each arm divided by the sum of flies in both arms. The average of two reciprocal experiments yielded the performance index (PI). For learning analyses, flies were tested immediately after a single training cycle. For LTM analyses, flies were trained with 5 cycles spaced at 15-min rest intervals, and tested 24 hr later; for LT-ARM analyses, flies were submitted to five massed conditioning cycles and memory was tested 24 hr later. Flies used for odor avoidance tests after electric shock and response to electric shock were treated as described by Pascual and Préat, 2001. For experiments involving neuronal blockade with shi^ts^*,* flies were transferred to preheated bottles in a 33°C room, immediately after the end of the last cycle of the training protocol. For experiments involving neuronal activation with dTrpA1, the conditioning tubes containing flies were plugged on a 31°C air flow at 2 L.min^−1^. Note that the behavior experiments in Figure 1B were conducted as previously described (Scheunemann et al., 2012). For cxm feeding, flies were transferred to vials containing filter paper soaked with 35 mM cxm in mineral water and 5% sucrose for 14–16 hr before training. After training and until memory test, flies were kept on regular food.

#### Immunohistochemistry Experiments

##### GFP Stainings

SPNsplit-Gal4: *SPNsplit-Gal4* female flies were crossed with UAS-mCD8::GFP. SPN-Gal4: *VT026326-Gal4; UAS-mCD8::GFP* and *GH298-Gal4* ;*UAS-mCD8::GFP* female flies were crossed to CS males. Prior to dissection, whole flies of female F1 progenies (3-4 days after eclosion at 25°C) were fixed in 4% formaldehyde in PBT (PBS containing 1% Triton X-100) at 4°C overnight. Brains were dissected in Drosophila Ringer solution and fixed for 1 hr at room temperature (RT) in 4% formaldehyde in PBT. Samples were then rinsed three times for 20 min in PBT, blocked with 2% bovine serum albumin in PBT for 2 hr, and incubated with rabbit anti-GFP (1:400; Invitrogen Molecular Probes) and mouse anti-nc82 (1:100; DSHB) primary antibodies in the blocking solution (4°C overnight). Brains were washed three times for 20 min in PBT and then incubated with anti-rabbit secondary antibody conjugated to Alexa Fluor 488 and anti-mouse Alexa Fluor 591 (1:400; Invitrogen Molecular Probes) in the blocking solution for 3 hr at RT. After three washes (20 min), brains were mounted in Prolong Mounting Medium (Life Technologies) for microscopy analysis. Images were acquired using a Nikon A1R confocal microscope. Confocal Z-stacks were acquired in 1.5-μm slices and imported into NIH ImageJ for analyses.

Intersection: *GH298-Gal4*; *UAS-mCD8::GFP* females were crossed to VT026326-Gal80 males.

5HT staining: *GH298-Gal4*; *UAS-mCD8::GFP* females were crossed to *UAS-Trh-RNAi1* or CS males.

Double staining: *30E11-LexA; VT026326-Gal4>UAS-tdTomato*; females were crossed to Aop-mCD8::GFP males and *NP0047-Gal4 ;VT026326-LexA* females were crossed to *UAS-RFP ;Aop*-*mCD8::GFP* males*.*

GRASP: *30E11-LexA ;VT026326-Gal4>UAS-tdTomato* females were crossed to UAS-GFP_sp11_/Gla ;Aop-GFP_sp1-10_/TM6_Tb,e_ males, and female F1 progeny excluding the balancer were selected. All histochemical experiments were conducted on F1 female brains according to the protocol described above. Additional primary antibodies used were: anti-5HT (Sigma, mouse, 1:200), anti-HA (Sigma/Roche, rat, 1:200), anti-dsRed (Clontech, rabbit, 1:200) and anti-GRASP (anti-GFP, Sigma, mouse 1:400). Additional secondary antibodies: anti-rat Alexa Fluor 591, anti-mouse Alexa Fluor 488 and anti-rabbit Alexa Fluor 591 (1:400; Invitrogen Molecular Probes).

#### 3D Reconstruction of Confocal Images Using Imaris

Drosophila whole-mount brain confocal Z-stacks of *SPNsplit-Gal4>UAS-mcd8::GFP* labeled with anti-GFP and anti-nc82 were imported into the Imaris program. The SPN was reconstructed using the Imaris “FilamentTracer” algorithm of the GFP channel in semi-automated mode from a manually pre-defined starting point. Brain neuropils were visualized using the classical Imaris 3D viewer of the nc82 channel.

#### In Vivo Calcium Imaging

*In vivo* confocal imaging and subsequent data analysis of spontaneous activity was performed following a previously described protocol (Plaçais et al., 2012). Images were acquired at a rate of one image every 427 ms. Only female flies were used in imaging experiments. Three 1000-image recordings were performed for each fly tested (except for the experiments detailed below). The activity of MP1 neurons was reported using the trace of the normalized fluorescence variations ΔF/F0 (expressed in %) from the mushroom body projections. For each time trace, the Fourier transform was calculated using the fft Matlab built-in function. The power spectrum was then calculated as the squared modulus of the Fourier Transform. Rhythmic spontaneous activity in the time domain resulted in a peak in the power spectrum that had a finite width, as oscillations are intrinsically noisy. A fit of a Lorentzian curve to the power spectrum was performed to yield an estimate of the central frequency of the peak, *f*_0_, and the width of the peak at half its maximal value, Δ*f*. *f*_0_ defined the characteristic frequency of the oscillation and frequency fluctuations around *f*_0_. When the Lorentzian fit failed to identify a peak in the power spectrum, we attribute it a 0 frequency, which corresponds to a signal with no oscillation. No flies were excluded from the analysis.

The calculation of the signal amplitude is as follows: to estimate the “floor” value of the signal, we extract all the datapoints in the interval between the lowest value and the 30% quartile, and we take the mean of these datapoints. Similarly, to estimate the “ceiling value”, we extract all the datapoints between the 70% quartile and the maximum value, and we calculate the mean of these datapoints. The amplitude is the difference between the ceiling and the floor values. See also Plaçais et al. (2012).

##### TrhRNAi Experiments

*tubGal80*^*ts*^*,30E11-LexA;VT026326-Gal4* females were crossed to *Aop-GCaMP3;UAS-Trh-RNAi1 or Aop-GCaMP3;UAS-Trh-RNAi2* males. The progeny were kept at 18°C throughout development and adult flies were placed at 30°C 3 days before the experiment in order to strongly induce RNAi expression. F1 female flies used for imaging were either naive or imaged within a timeframe of 30-90 min after spaced or massed conditioning. Dissection and *in vivo* calcium imaging were conducted as described above.

##### 5HT-2A-RNAi Experiments

*tubGal80*^*ts*^*;NP0047* females were crossed to *UAS-GCaMP3;UAS-5HT-2A-RNAi1 or UAS-GCaMP3;UAS-5HT-2A-RNAi2* males. F1 female flies used for imaging were either naive or analyzed within a timeframe of 30-90 min after spaced or massed conditioning. Dissection and *in vivo* calcium imaging were conducted as described above.

##### dnc-RNAi Experiments

*dncRNAi;Aop-GCaMP3;* females were crossed to *tubGal80*^*ts*^*,30E11-LexA;VT026326-Gal4* males. The progeny were kept at 30°C throughout development in order to strongly induce RNAi expression. F1 female flies used for imaging were naive. Dissection and *in vivo* calcium imaging were conducted as described above.

##### Shibire Experiments

*VT026326-LexA;;NP0047-Gal4* females were crossed to *UAS-GCaMP3;Aop-shi*^*ts*^ males. F1 female flies were dissected as described above, with the exception that they were mounted on a heating cell in order to calibrate the temperature of the Drosophila Ringer’s solution used for brain perfusion. Images were acquired at a rate of one image every 125 ms, and one 1000-image recording was performed at the permissive temperature (25°C). Subsequently, the temperature of the heating cell was shifted to 33°C to heat the Ringer’s solution to the restrictive temperature. After 20 min, another 1000-image recording was taken at a rate of one image every 125 ms.

##### TrpA1 Experiments

*VT026326-LexA;;NP0047* females were crossed to *UAS-GCaMP3;Aop-TrpA1* males. The progeny were kept at 23°C throughout development to avoid TrpA1 expression, while allowing for detectable GCaMP3 expression. F1 female flies were dissected as described above and mounted as described for *shibire* experiments. Images were acquired at a rate of one image every 125 ms, and two 750-image recordings were performed at the non-activating temperature (20°C). Subsequently, the temperature of the heating cell was shifted to 30°C in order to heat the Ringer’s solution to the activating temperature. Immediately after this temperature shift, two 750-image recordings were performed at a rate of one image every 125 ms. Because of the sensitivity of the TrpA1 channel, acquisition times were reduced for this experiment in order to restrict exposure to the activating temperature. Instead, the mean of two replicate recordings was used for each temperature regime.

#### In Vivo PKA Imaging

*In vivo* two-photon imaging of PKA activity using the AKAR2 sensor was performed according to a previously described protocol (Gervasi et al., 2010), with the following modifications. Flies homozygous for both VT057280 and UAS-AKAR2 were used in order to obtain sufficient signal for 2-photon FRET imaging. For RNAi experiments, F1 females were used from the crossing of *Dnc*^*RNAi*^*;VT57280,UAS-AKAR2* females with *VT57280,UAS-AKAR2* males. For experiments in the *dnc*^*1*^ background, *+;VT57280,UAS-AKAR2* males were compared to F1 males obtained from the crossing of *dnc*^*1*^*;VT57280,UAS-AKAR2* females with *VT57280,UAS-AKAR2* males. All flies were raised at 25°C and kept at 30°C for 2 days before experiments. Images were acquired on a Leica TCS-SP5 microscope. The AKAR2 sensor was excited at 850 nm using a tunable MaiTai DeepSee pulsed laser (Spectra Physics), through a 25x water-immersion objective (HCX IRAPO, NA 0.95). Images were acquired in a plane showing projections of the SPN neuron on the peduncle region at a rate of 1 image every 5 s. Stocks of IBMX or forskolin (Sigma-Aldrich) were dissolved and aliquoted in DMSO at 200 mM and 14 mM, respectively, and subsequently diluted 100 times in Drosophila Ringer’s solution on the day of the experiment. 10 μL of this solution were injected into the 90-μL droplet of Drosophila Ringer’s solution bathing the fly brain during image acquisition, resulting in a final concentration of 200 μM for IBMX and 14 μM for forskolin. Image analysis was performed using a custom-written MATLAB program. ROIs were manually delimited around SPN projections in each hemisphere, and at each time point the average intensities of YFP and CFP channels were calculated, background-subtracted and divided to obtain the FRET ratio. FRET ratio time traces were normalized to a baseline value calculated from the 60 s preceding drug application. Plateau responses were measured as the average normalized FRET ratio starting 60 s after drug application and extending over a 120-s duration.

#### Measurements of Locomotor Activity

Two- to three-day old flies were selected 72 hr before the assay and placed in regular food vials at 30°C (RNAi induction) or 18°C (shibire). In each experiment, 32 female flies were assayed. Each fly was transferred to Trikinetics (http://www.trikinetics.com/) glass tubes sealed with fly food on one side and a plug on the other side. Tubes were placed in Trikinetics Drosophila Activity Monitors the evening prior to activity recordings. Monitors were housed in a temperature-controlled incubator at 25°C under a 12 hr/12 hr light-dark cycle, and locomotor activity was recorded the following morning. For shibire experiments, 1 hr before the onset of recording the incubator was set to 33°C and subsequently maintained at this temperature throughout the experiment. Beam breaks were recorded at 1-min intervals. Activity scores were calculated by averaging the total number of beam breaks during a 3-hr period after conditioning.

#### Cloning

All cloning was performed using the Gateway Technology (Life Technologies), following the manufacturer’s instructions. A 2.1 kb PCR fragment resulting from the amplification of the Vienna Tile GAL4 driver line VT026326 (Vienna Drosophila Resource Center) was cloned into the pBPGAL80Uw-6 vector for *VT026326-Gal80*^*+*^, and into the pBPnlsLexA::p65Uw vector for *VT026326-LexA* (Pfeiffer et al., 2010). To generate the *SPNsplit-Gal4*, the same PCR fragment was cloned into the apBPp65ADZpUw vector and a 2.1 kb PCR fragment resulting from the amplification of the Vienna Tile GAL4 driver line VT057280 (Vienna Drosophila Resource Center) was cloned into the pBPZpGAL4DBDUw vector (Pfeiffer et al., 2010). To amplify VT026326 sequences we used the following primers: Forward, 5′-GGGGACAAGTTTGTACAAAAAAGCAGGCTCGGACCACATTAAAATCAC-3′ and Reverse, 5′GGGGACCACTTTGTACAAGAAAGCTGGGTGCTGCCAGATGATGGCCC-3′. To amplify VT057280 sequences we used: Forward, 5′-GGGGACAAGTTTGTACAAAAAAGCAG

GCTTAATAGGAACTGCAGAACG-3′ and Reverse, 5′-GGGGACCACTTTGTACAAGAA

AGCTGGGTTGCAGGCGCAATTTTCAATG-3′. The Vienna Tiles-specific sequences are underlined. Vectors were verified by restriction. Transgenic fly strains were obtained by site-specific embryonic injection of the resulting vectors, which was outsourced to Rainbow Transgenic Flies (CA, USA). Insertion sites were VK00027 (3^rd^ chromosome) for *VT026326-Gal80* and attP18 (X-chromosome) for *VT026326-LexA.* For *SPNsplit-Gal4*, the insertion sites were attp40 (AD, 2^nd^ chromosome) and attp2 (BD, 3^rd^ chromosome).

### Quantification and Statistical Analysis

For behavioural experiments, 2 groups of about 40 flies were conditioned and tested for the two odors used in this study reciprocally. From these two groups the performance index was calculated as previously described, which represents a n of 1 (Pascual and Préat, 2001). For imaging experiments, a n of 1 corresponds to one fly brain. All data are presented as mean ± SEM. Comparisons of the data series between two conditions were achieved by a two-tailed unpaired or paired t test as indicated. Comparisons between more than two distinct groups were made using a one-way ANOVA, followed by Newman-Keuls pairwise comparisons between the experimental groups and their controls. ANOVA results are presented as the value of the Fisher distribution *F*_(*x,y*)_ obtained from the data, where *x* is the number of degrees of freedom between groups and *y* is the total number of degrees of freedom for the distribution. Statistical tests were performed using the GraphPad Prism 5 software. In the figures, asterisks illustrate the significance level of the t test, or of the least significant pairwise comparison following an ANOVA, with the following nomenclature: ^∗^p < 0.05; ^∗∗^p < 0.01; ^∗∗∗^p < 0.001; NS: not significant, p > 0.05).

## Supplemental Information

Document S1. Figures S1–S8 and Table S1

Video S1. Whole-Brain Labeling of the SPN Driver GH298-Gal4 (1.1), VT026326-Gal4 (1.2), and Their Intersection (1.3), Related to Figure 1 Video 1.1 displays whole-brain expression of GH298-Gal4 driving mcd8::GFP (green) and anti-nc82 neuropil counterstaining (magenta). Video 1.2 displays whole-brain expression of VT026326-Gal4 driving mcd8::GFP (green) and anti-nc82 neuropil counterstaining (magenta). Video 1.3 displays whole-brain expression of the genetic intersection between VT026326-Gal80^+^ and GH298-Gal4 driving mcd8::GFP (green) and anti-nc82 neuropil counterstaining (magenta). This demonstrates that SPN expression was excluded from GH298-Gal4 labeling, whereas the remaining expression pattern resembles the normal GH298-Gal4 pattern.

Video S2. Whole-Brain Labeling of the SPNsplit-Gal4, Related to Figure 2 Video displaying whole-brain expression of SPNsplit-Gal4 driving mcd8::GFP (green) and anti-nc82 neuropil counterstaining (magenta). The expression pattern is exclusively restricted to the SPN.

Video S3. 3D Reconstruction of the SPN, Related to Figure 2 3D reconstruction of the SPN in SPNsplit-Gal4>UAS-mcd8::GFP-labeled brains, with anti-nc82 neuropil counterstaining to visualize brain regions.

Video S4. Double Labeling of SPN and MP1 at the MB Peduncle (Driver Combination 1), Related to Figure 4 Video displaying SPN expression at the level of the MB peduncle using VT026326-LexA>Aop-mCD8::GFP (magenta), with MP1 expression using NP0047-Gal4>UAS-RFP (green). The SPN projections co-localize with the MP1 projections covering the posterior part of the MB peduncle.

Video S5. Double Labeling of SPN and MP1 at the MB Peduncle (Driver Combination 2), Related to Figure 4 Video displaying SPN expression at the level of the MB peduncle using VT026326-Gal4>UAS-tdTomato (magenta), with MP1 expression using 30E11-LexA>Aop-mCD8::GFP (green). The SPN projection co-localizes with the MP1 projections covering the posterior part of the MB peduncle. The expression intensity of 30E11 is weaker than NP0047-Gal4, resulting in a smaller overlap of projection sites for this combination of drivers.

Video S6. Labeling of the SPN and GRASP Signals with MP1 at the MB Peduncle, Related to Figure 4 Video displaying SPN expression at the level of the MB peduncle using VT026326>UAS-tdTomato (magenta) and GRASP signals, showing reconstituted GFP between VT026326-Gal4 (SPN) and 30E11 (MP1). The GRASP signals specifically localize on the SPN projection surrounding the MB peduncle.

Document S1. Article plus Supplemental Information
