# Supplementary material for: Dunce Phosphodiesterase Acts as a Checkpoint for Drosophila Long-Term Memory in a Pair of Serotonergic Neurons
Source: Neuron. 2018 Apr 18;98(2):350–365.e5. doi: 10.1016/j.neuron.2018.03.032 (PMC5919781; doi:10.1016/j.neuron.2018.03.032)
Supplement: Document S1. Figures S1–S8 and Table S1 [file mmc1.pdf]

Neuron, Volume *98*

Supplemental Information

Dunce Phosphodiesterase Acts as a Checkpoint for Drosophila Long-Term Memory in a Pair of Serotonergic Neurons

Lisa Scheunemann, Pierre-Yves Plaçais, Yann Dromard, Martin Schwärzel, and Thomas Preat

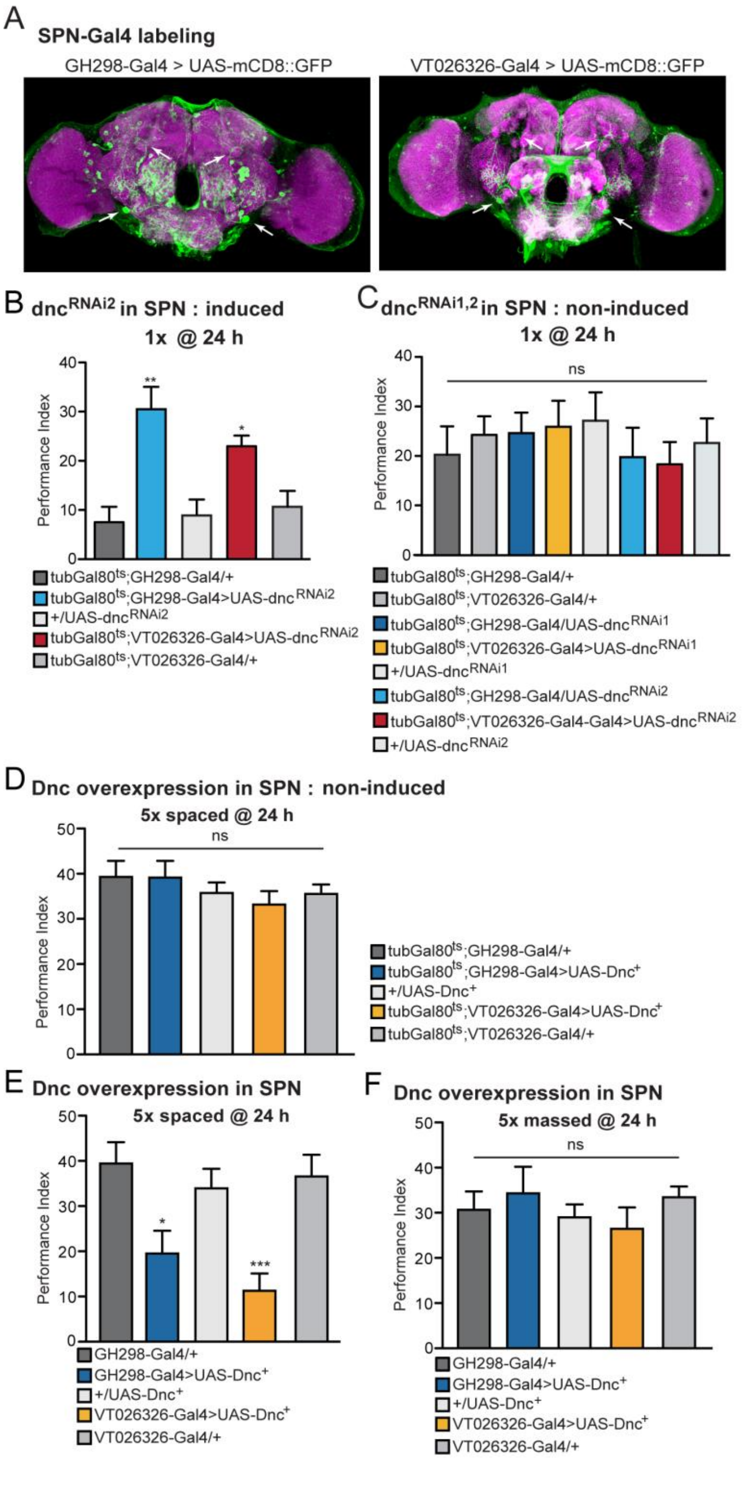

A
SPN-Gal4 labeling
GH298-Gal4 > UAS-mCD8::GFP
VT026326-Gal4 > UAS-mCD8::GFP
B dncRNAi2 in SPN : induced
1x @ 24 h
Performance Index
tubGal80ts;GH298-Gal4/+
tubGal80ts;GH298-Gal4>UAS-dncRNAi2
+/UAS-dncRNAi2
tubGal80ts;VT026326-Gal4>UAS-dncRNAi2
tubGal80ts;VT026326-Gal4/+
C dncRNAi1,2 in SPN : non-induced
1x @ 24 h
ns
Performance Index
tubGal80ts;GH298-Gal4/+
tubGal80ts;VT026326-Gal4/+
tubGal80ts;GH298-Gal4/UAS-dncRNAi1
tubGal80ts;VT026326-Gal4>UAS-dncRNAi1
+/UAS-dncRNAi1
tubGal80ts;GH298-Gal4/UAS-dncRNAi2
tubGal80ts;VT026326-Gal4-Gal4>UAS-dncRNAi2
+/UAS-dncRNAi2
D Dnc overexpression in SPN : non-induced
5x spaced @ 24 h
ns
Performance Index
tubGal80ts;GH298-Gal4/+
tubGal80ts;GH298-Gal4>UAS-Dnc+
+/UAS-Dnc+
tubGal80ts;VT026326-Gal4>UAS-Dnc+
tubGal80ts;VT026326-Gal4/+
E Dnc overexpression in SPN
5x spaced @ 24 h
Performance Index
GH298-Gal4/+
GH298-Gal4>UAS-Dnc+
+/UAS-Dnc+
VT026326-Gal4>UAS-Dnc+
VT026326-Gal4/+
F Dnc overexpression in SPN
5x massed @ 24 h
ns
Performance Index
GH298-Gal4/+
GH298-Gal4>UAS-Dnc+
+/UAS-Dnc+
VT026326-Gal4>UAS-Dnc+
VT026326-Gal4/+

Figure S1: Supplemental information for Figure 1. A GH298-Gal4 expression was visualized with UAS-mCD8::GFP, which labels a pair of projection neurons located in the gnathal ganglia (GNG, arrowhead) that project to the superior clamp (SCL) surrounding the MB peduncle (arrowhead). VT026326-Gal4 was identified driving identical expression in the SPN (arrowhead), as shown by VT026326>UAS-mCD8::GFP labeling. **B** Expression of a second non-overlapping RNAi targeting Dnc using the thermo-inducible drivers tub-Gal80^{ts};GH298 and tub-Gal80^{ts};VT026326 results in significantly increased memory scores at 24 h, after 1 training cycle ($F_{4/56} = 4,7$, $p = 0.01$, $n \geq 9$). **C** Non-induced controls for the Dnc knockdown experiment: flies kept at 18°C before the experiment showed no difference in memory performance compared to the genotypic controls ($F_{7/56} = 0.6$, $p = 0.69$, $n \geq 8$). **D** Non-induced controls for the Dnc overexpression experiment: flies kept at 18°C before the experiment showed no difference in memory performance compared to the genotypic controls ($F_{4/30} = 1.08$, $p < 0.87$, $n \geq 7$). **E** Constitutive overexpression of Dnc in the SPN impairs LTM using GH298-Gal4 ($F_{2/29} = 4.81$, $p = 0.016$, $n \geq 9$) and VT026326-Gal4 ($F_{2/29} = 10.51$, $p < 0.001$, $n \geq 9$). **F** Memory performances after 5x massed cycles were not affected by Dnc overexpression in the SPN ($F_{4/42} = 0.59$, $p = 0.66$, $n \geq 8$). Data are presented as mean ± SEM. $^{*}p < 0.05$; $^{**}p < 0.01$; $^{***}p < 0.0001$, ns = not significant. Statistical tests were performed using one-way ANOVA. Stars indicate the least significance level in a Newman-Keuls post-hoc comparison of indicated groups.

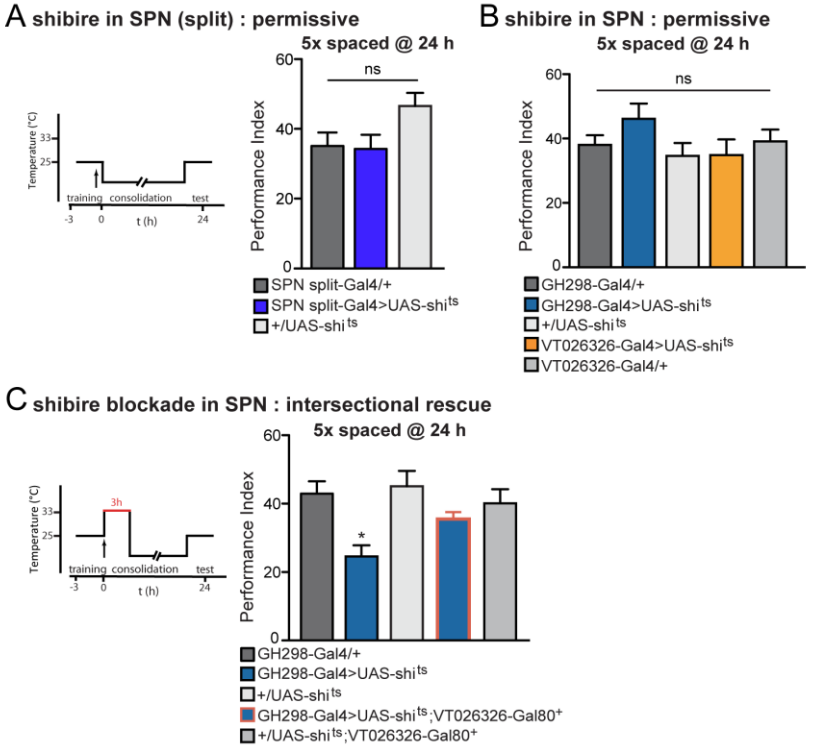

Figure S2: Supplemental information for Figure 2. A SPNsplit-Gal4>UAS-shi^{ts} did not show any difference in memory performance as compared to the genotypic controls when the experiment was conducted at the permissive temperature (20°C) ($F_{2/27} = 2,3$, $p = 0.11$, $n \geq 9$). **B** GH298-Gal4>UAS-shi^{ts} and VT026326-Gal4>UAS-shi^{ts} did not show any impairment in memory performance at the permissive temperature (20°C) ($F_{4/46} = 1.21$, $p = 0.31$, $n \geq 8$). **C** The genetic intersection between VT026326-Gal80^{+} and GH298, which turns-off Gal4 expression in the SPN, rescues LTM in flies expressing UAS-shi^{ts}. GH298-Gal4>UAS-shi^{ts},VT026326-Gal80 flies and their genotypic controls displayed significantly higher memory performances than GH298-Gal4>UAS-shi^{ts} flies ($F_{4/44} = 4.2$, $p = 0.005$, $n \geq 8$). The performance of GH298-Gal4>UAS-shi^{ts},VT026326-Gal80 flies was statistically indistinguishable from the genotypic controls according to pairwise post-hoc comparisons. Data are presented as mean + SEM. *$p < 0.05$; **$p < 0.01$; ***$p < 0.0001$, ns = not significant. Statistical tests were performed using one-way ANOVA. Stars indicate the least significance level in a Newman-Keuls post-hoc comparison of indicated groups.

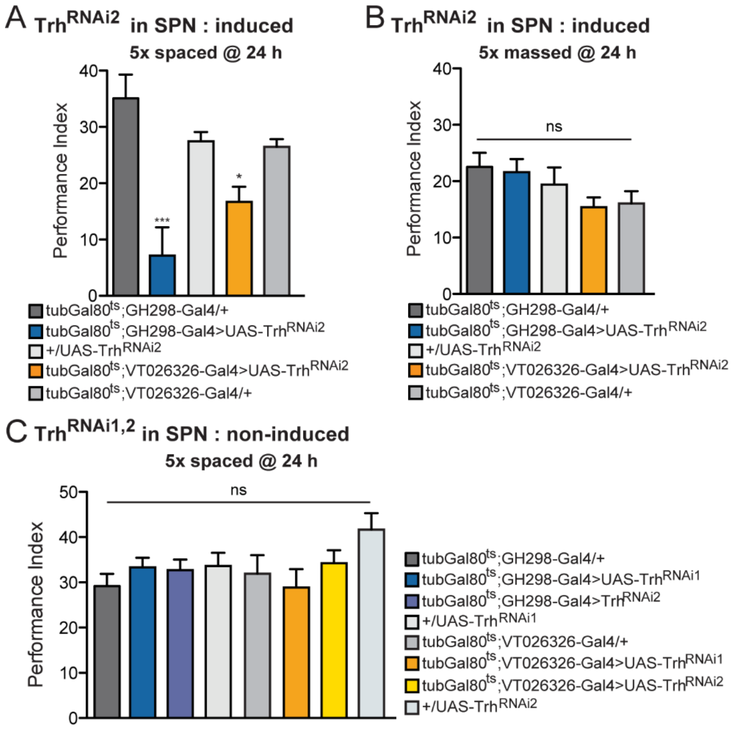

Figure S3: Supplemental information for Figure 3. A Induced knockdown of Trh in the SPN using a second RNAi impaired LTM formation after 5x spaced training in tub-Gal80^{ts};GH298>UAS-Trh^{RNAi2} ($F_{2/27} = 14.13$, $p < 0.0001$, $n \geq 8$) and tub-Gal80^{ts};VT026326>UAS-Trh^{RNAi2} ($F_{2/32} = 32.45$, $P < 0.0001$, $N \geq 8$) flies. **B** Memory performance at 24 h after 5x massed training was not impaired by knockdown of Trh in the SPN with Trh^{RNAi2}. Memory scores of tub-Gal80^{ts};GH298>UAS-Trh^{RNAi2} and tub-Gal80^{ts};VT026326>UAS-Trh^{RNAi2} flies did not differ from their respective controls ($F_{4/42} = 1.75$, $p = 0.16$, $n \geq 7$). **C** Non-induced controls of the Trh knockdown experiment: all genotypes, kept at 18°C before the experiment, showed similar memory performance ($F_{7/60} = 1.45$, $p = 0.19$, $n \geq 7$). Data are presented as mean + SEM. *$p < 0.05$; **$p < 0.01$; ***$p < 0.0001$, ns = not significant. Statistical tests were performed using one-way ANOVA. Stars indicate the least significance level in a Newman-Keuls post-hoc comparison of indicated groups.

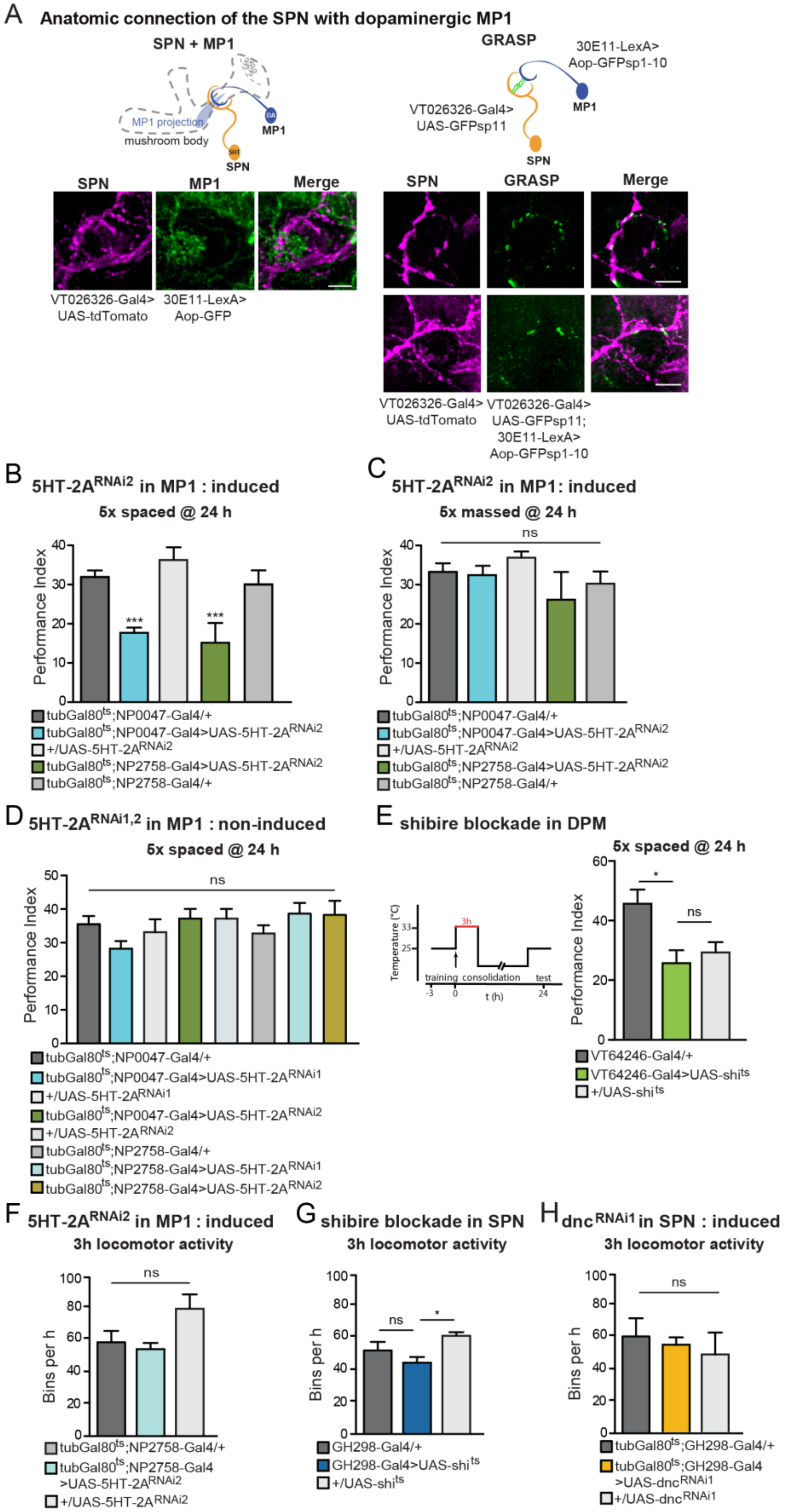
A Anatomic connection of the SPN with dopaminergic MP1
SPN + MP1
MP1 projection
mushroom body
MP1
SPN
GRASP
30E11-LexA>
Aop-GFPsp1-10
VT026326-Gal4>
UAS-GFPsp11
SPN
MP1
Merge
GRASP
VT026326-Gal4>
UAS-tdTomato
30E11-LexA>
Aop-GFP
VT026326-Gal4>
UAS-tdTomato
VT026326-Gal4>
UAS-GFPsp11;
30E11-LexA>
Aop-GFPsp1-10
B 5HT-2ARNAi2 in MP1 : induced
5x spaced @ 24 h
Performance Index
tubGal80ts;NP0047-Gal4/+
tubGal80ts;NP0047-Gal4>UAS-5HT-2ARNAi2
+/UAS-5HT-2ARNAi2
tubGal80ts;NP2758-Gal4>UAS-5HT-2ARNAi2
tubGal80ts;NP2758-Gal4/+
C 5HT-2ARNAi2 in MP1: induced
5x massed @ 24 h
ns
D 5HT-2ARNAi1,2 in MP1 : non-induced
5x spaced @ 24 h
ns
tubGal80ts;NP0047-Gal4>UAS-5HT-2ARNAi1
+/UAS-5HT-2ARNAi1
tubGal80ts;NP2758-Gal4>UAS-5HT-2ARNAi1
E shibire blockade in DPM
Temperature (°C)
3h
training
consolidation
test
t (h)
VT64246-Gal4/+
VT64246-Gal4>UAS-shits
+/UAS-shits
F 5HT-2ARNAi2 in MP1 : induced
3h locomotor activity
Bins per h
tubGal80ts;NP2758-Gal4
>UAS-5HT-2ARNAi2
G shibire blockade in SPN
3h locomotor activity
GH298-Gal4/+
GH298-Gal4>UAS-shits
H dncRNAi1 in SPN : induced
3h locomotor activity
tubGal80ts;GH298-Gal4/+
tubGal80ts;GH298-Gal4
>UAS-dncRNAi1
+/UAS-dncRNAi1

Figure S4: Supplemental information for Figure 4. A SPN (green) and MP1 (magenta) were simultaneously visualized using VT026326-Gal4>UAS-tdTomato and 30E11-LexA>Aop-mCD8::GFP, respectively. GFP Reconstitution Across Synaptic Partners (GRASP) showing reconstituted GFP signals (green) at the level of the SPN projection around the MB peduncle in two additional examples. Scale bar: 5 µm. **B** Induced knockdown of the serotonergic receptor 5HT-2A at the adult stage in MP1 neurons using a second RNAi impaired LTM using tub-G80^{ts};NP0047 ($F_{2/29} = 8.72$, $p = 0.001$, $n \geq 9$) and tub-G80^{ts};NP2758 ($F_{2/28} = 8.15$, $p = 0.002$, $n \geq 9$). **C** Induced knockdown of the serotonergic receptor 5HT-2A at the adult stage in MP1 using a second RNAi after massed training did not impair LT-ARM scores ($F_{4/52} = 0.65$, $p = 0.52$, $n \geq 8$). **D** Non-induced control flies for the 5HT-2A knockdown experiment: flies kept at 18°C before the experiment showed normal memory performance ($F_{8/72} = 0.89$, $p < 0.52$, $n \geq 7$). **E** DPM blockade in VT64246-Gal4>UAS-shi^{ts} after 5x spaced training was significantly different from VT64246-Gal4/+ but did not affect LTM performances in comparison to UAS-shi^{ts}/+. **F** Locomotor activity in flies with induced 5HT-2A knockdown in MP1 measured during 3 h using the Trikinetics assay was normal ($F_{2/9} = 3.8$, $p < 0.08$, $n = 3$). **G** Locomotor activity after SPN blockade in GH298-Gal4>UAS-shi^{ts} flies was different from +/UAS-shi^{ts}, but not GH298-Gal4/+ ($F_{2/9} = 7{,}9$, $p = 0.02$, $n = 3$). **H** Dnc knockdown in the SPN did not alter locomotor activity in the flies ($F_{2/9} = 3.43$, $p = 0.06$, $n = 3$). Data are presented as mean + SEM. *p < 0.05; **p < 0.01; ***p < 0.0001, ns = not significant. Statistical tests were performed using one-way ANOVA. Stars indicate the least significance level in a Newman-Keuls post-hoc comparison of indicated groups.

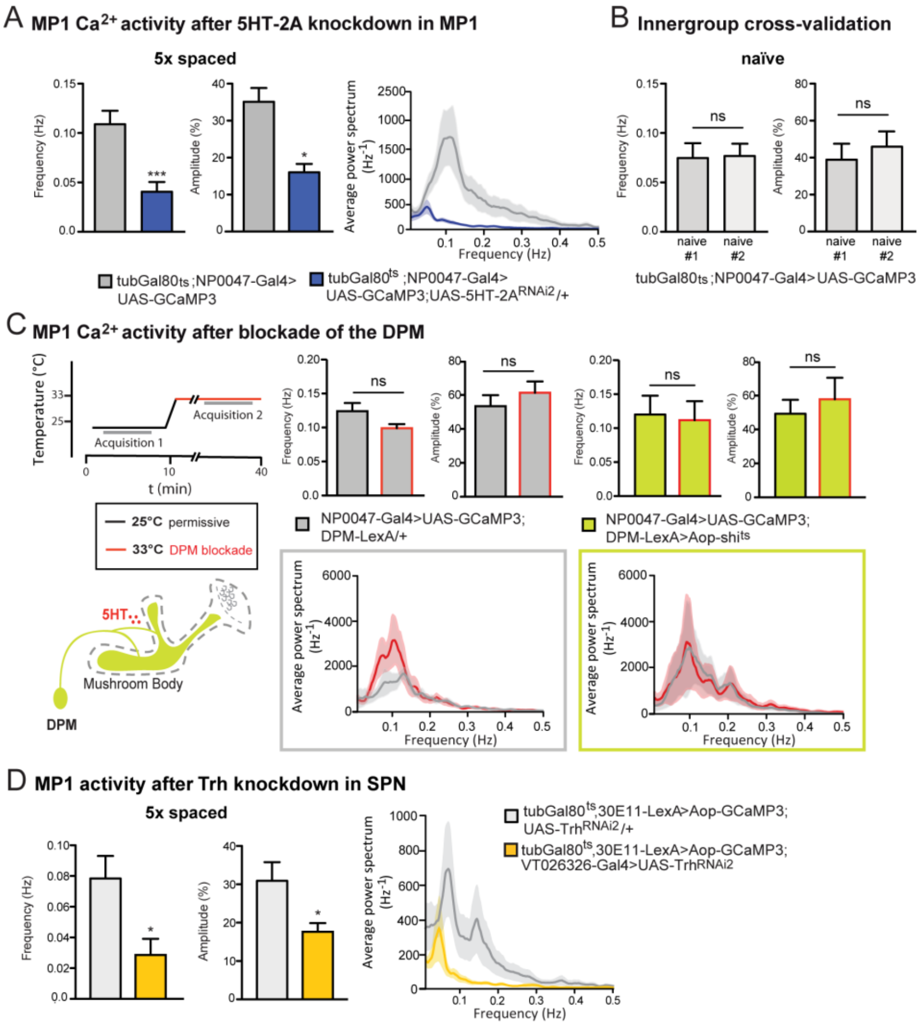

Figure S5: Supplemental information for Figure 5. A Knockdown of the 5HT-2A receptor in MP1 using a second RNAi resulted in decreased Ca^{2+} activity in MP1 neurons and a loss of the oscillatory pattern after 5x spaced training in comparison to control flies (frequency: *t* test, $t_{11} = 6.8$, $p < 0.001$; amplitude: *t* test, $t_{11} = 2.4$, $p = 0.031$, $n = 6$). Power spectra are shown for each genotype. **B** Comparing the randomly generated subdivisions group #1 and group #2 of all calcium traces from naïve flies resulted no significant effects of MP1 activity (frequency: *t* test, $t_{24} = 0.09$, $p = 0.92$; amplitude: *t* test, $t_{24} = 0.73$, $p = 0.46$, $n = 24$). **C** Blockade of synaptic transmission from the DPM using Aop-shi^{ts} did not alter MP1 Ca^{2+} activity (frequency: *t* test, $t_{10} = 0.21$, $p = 0.83$; amplitude: *t* test, $t_{10} = 0.56$, $p = 0.58$, $n = 6$) in comparison to the genotypic control flies (frequency: *t* test, $t_8 = 1.8$, $p = 0.1$; amplitude: *t* test, $t_8 = 0.18$, $p = 0.42$, $n = 6$). Power spectra are shown for each genotype and condition. **D** Knockdown of the Trh receptor in SPN using a second RNAi shows decreased Ca^{2+} activity in MP1 after 5x spaced training in comparison to control flies (frequency: *t* test, $t_{11} = 12.8$, $p = 0.017$; amplitude: *t* test, $t_{11} = 2.6$, $p = 0.024$, $n = 6$). Power spectra are

shown comparing frequency bands for each genotype. Data are presented as mean ± SEM. *$p < 0.05$; **$p < 0.01$; ***$p < 0.0001$, ns = not significant.

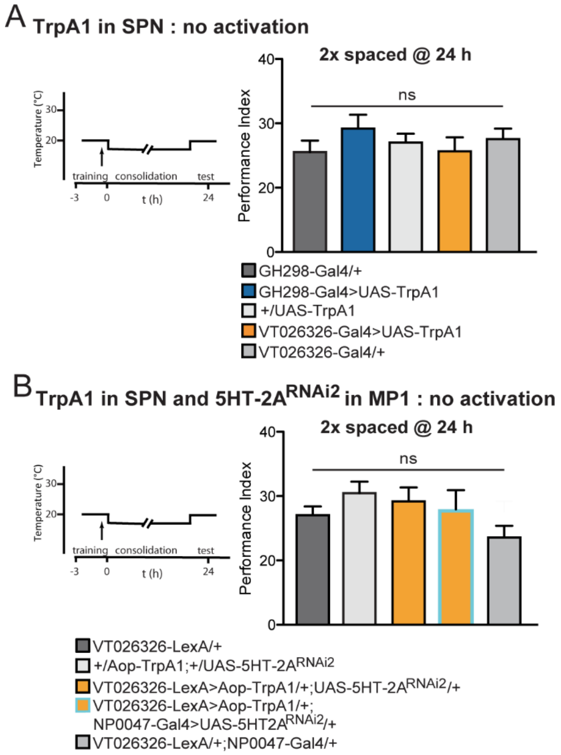

Figure S6: Supplemental information for Figure 6. A GH298-Gal4>UAS-TrpA1 and VT026326-Gal4>UAS-TrpA1 flies exhibited normal memory performance when the experiment was conducted at the non-activating temperature (20°C) ($F_{4/46}$ = 1.21, p = 0.31, n ≥ 8). **B** Likewise, flies expressing VT026326-LexA>AopTrpA1;NP0047-Gal4/+ and VT026326-LexA>AopTrpA1;NP0047-Gal4>UAS-5HT-2A^{RNAi} exhibited normal memory performance when the experiment was conducted at the non-activating temperature (20°C) ($F_{4/40}$ = 1.75, p = 0.16, n ≥ 8). Data are presented as mean ± SEM. *p < 0.05; **p < 0.01; ***p < 0.0001, ns = not significant. Statistical tests were performed using one-way ANOVA. Stars indicate the least significance level in a Newman-Keuls post-hoc comparison of indicated groups.

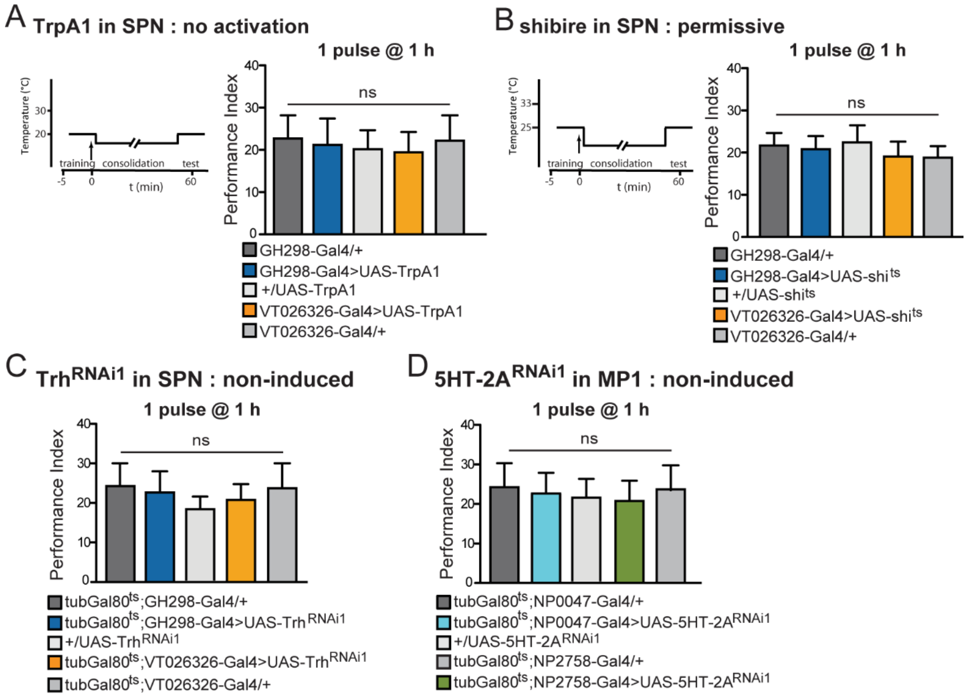

Figure S7: Supplemental information for Figure 7. A Expressing UAS-TrpA1 in the SPN does not affect memory performance after 1-pulse training at 1 h, when the experiment is conducted at low temperature ($F_{4/35}$ = 0.3, p = 0.83, n = 7). **B** Flies expressing UAS-shi^{ts} in the SPN exhibit normal 1-h memory after 1-pulse training at the permissive temperature ($F_{4/35}$ = 0.27, p = 0.81, n = 7). **C** Non-induced controls for Trh^{RNAi} expression in the SPN *($F_{4/35}$ = 0.9, p = 0.7, n = 8).* **D** Non-induced controls for 5HT-$2A^{RNAi}$ expression in MP1 neurons *($F_{4/35}$ = 1.1, p = 0.8, n = 8).* Data are presented as mean ± SEM. *p < 0.05; **p < 0.01; ***p < 0.0001, ns = not significant. Statistical tests were performed using one-way ANOVA. Stars indicate the least significance level in a Newman-Keuls post-hoc comparison of indicated groups.

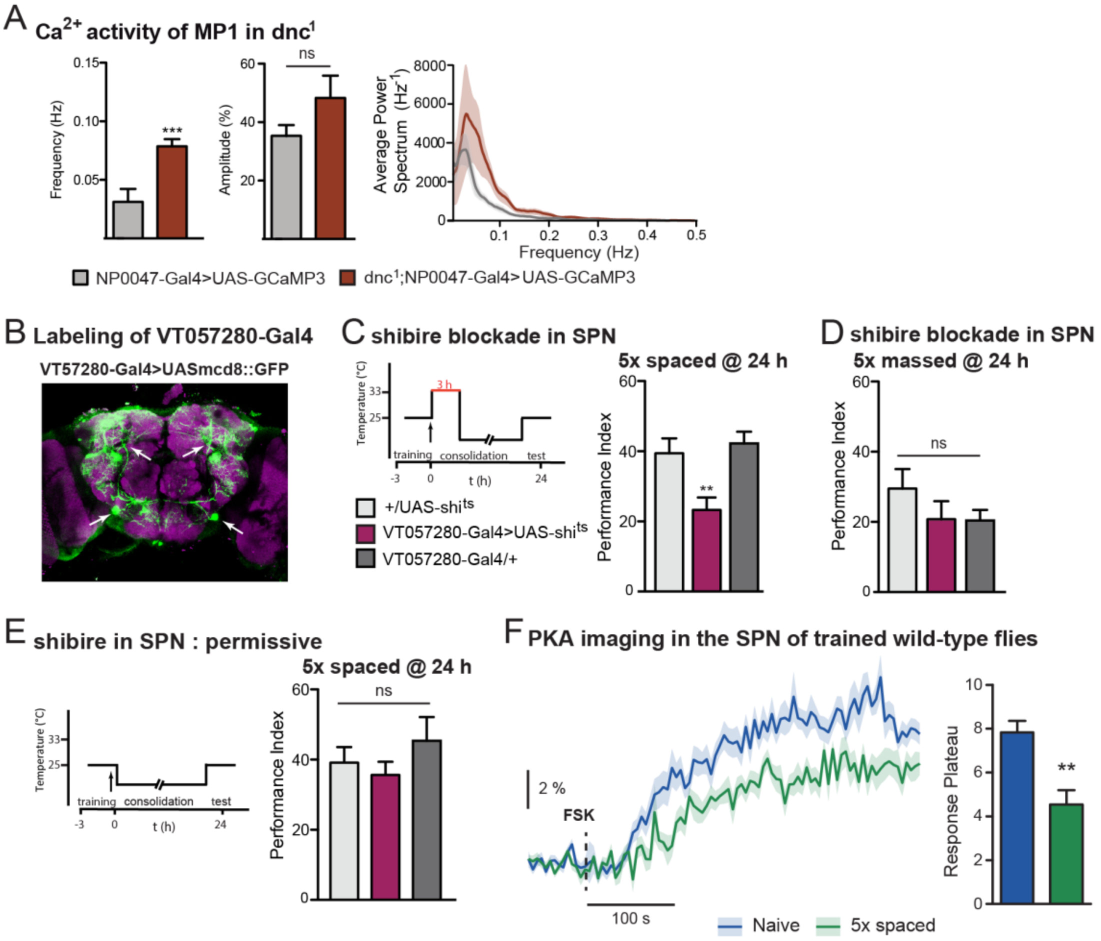

Figure S8: Supplemental information for Figure 8. A Naïve *dnc*[1] mutant flies displayed enhanced calcium oscillations in MP1 neurons in comparison to the genotypic control flies (frequency: *t* test, $t_{14} = 6.9$, $p < 0.0001$; amplitude: *t* test, $t_{14} = 2.1$, $p = 0.051$, $n = 9$). **B** VT057280>UAS-mCD8::GFP labeling confirmed that VT057280-Gal4 is capable of driving expression in the SPN (white arrowheads). **C** The behavioral phenotype was confirmed via blockage by shi^{ts}. Flies in which neuronal transmission was blocked for 3 h after conditioning showed impaired memory performance at 24 h ($F_{2/30} = 7{,}43$, $p = 0.0027$, $n \geq 10$). **D** After 5x massed training, SPN blockage during consolidation did not affect 24-h memory ($F_{2/24} = 1.2$, $p = 0.31$, $n \geq 8$). **E** After 5x spaced training, no differences in memory performance were observed at 24 h when RNAi was not induced ($F_{2/24} = 0.92$, $p = 0.41$, $n \geq 8$). **F** After 5x spaced training, forskolin treatment was still able to induce a PKA activation that was detectable by the AKAR2 sensor. The magnitude of the forskolin-induced activation was reduced compared to naïve flies (*t* test, $t_{19} = 3.9$, $p = 0.001$, $n = 10;11$). Data are presented as mean + SEM. *$p < 0.05$; **$p < 0.01$; ***$p < 0.0001$, ns = not significant. Statistical tests were performed using one-way ANOVA. Stars indicate the least significance level in a Newman-Keuls post-hoc comparison of indicated groups.

Genotype	Shock reactivity	Oct	MCH
UAS-Dnc^{+}/+	53.27 ± 7.18	55.14 ± 5.99	47.06 ± 7.18
VT026326-Gal4/+	60.1 ± 5.86	40.69 ± 6.58	58.85 ± 7.95
VT026326-Gal4/ UAS-Dnc^{+}	49.49 ± 5.72	31.57 ± 6.68	56.96 ± 7.39
GH298-Gal4/+	53.73 ± 7.34	36.55 ± 3.64	54.95 ± 9.07
GH298-Gal4/ UAS-Dnc^{+}	41.49 ± 6.74	54.2 ± 6.99	46.03 ± 12.39
UAS-Trh^{RNAi1}/+	61.19 ± 7.76	47.53 ± 4.94	51.36 ± 3.75
tubG80^{ts};VT026326-Gal4/+	70.07 ± 6.18	47.99 ± 5.88	56.87 ± 5.76
tubG80^{ts};VT026326-Gal4/UAS-Trh^{RNAi1}	58.56 ± 6.82	52.28 ± 4.6	50.54 ± 3.75
tubG80^{ts};GH298-Gal4/+	67.76 ± 5.13	57.82 ± 6.04	59.46 ± 7.47
tubG80^{ts};GH298-Gal4/ UAS-Trh^{RNAi1}	66.44 ± 5.74	44.22 ± 4.11	45.72 ± 6.98
UAS-Trh^{RNAi2}/+	60.41 ± 3.46	53.28 ± 5.2	59.25 ± 8.18
tubG80^{ts};VT026326-Gal4/ UAS-Trh^{RNAi2}	66.67 ± 4.96	51.51 ± 5.68	59.46 ± 7.47
tubG80^{ts};GH298-Gal4/ UAS-Trh^{RNAi2}	51.75 ± 7.2	44.64 ± 4.61	56.27 ± 7.34
UAS-5HT-2A^{RNAi1}/+	70.43 ± 4.59	57.08 ± 6.83	56.02 ± 6.6
tubG80^{ts};NP0047-Gal4/+	51.84 ± 6.78	49.29 ± 4.01	48.56 ± 7
tubG80^{ts};NP0047-Gal4/ UAS-5HT2A^{RNAi1}	51.84 ± 6.78	49.29 ± 4.01	57.15 ± 6.34
tubG80^{ts};NP2758-Gal4/+	60.25 ± 4.53	52.88 ± 5.37	49.38 ± 6.58
tubG80^{ts};NP2758-Gal4/ UAS-5HT2A^{RNAi1}	62.81 ± 4.58	59.13 ± 6.97	50.0 ± 5.62
UAS-5HT-2A^{RNAi2}/+	57.12 ± 3.44	48.15 ± 7.66	49.78 ± 5.25
tubG80^{ts};NP0047-Gal4/ UAS-5HT2A^{RNAi2}	37.42 ± 9.15	55.81 ± 5.52	41.61 ± 8.18
tubG80^{ts};NP2758-Gal4 / UAS-5HT2A^{RNAi2}	61.63 ± 5.6	49.93 ± 6.93	46.63 ± 4.83

Table S1: Supplemental olfactory acuity and shock response data for Figures 1, 3 and 4. $Gal80^{ts}$;GH298>UAS-dnc^{+} and $Gal80^{ts}$;VT026326>UAS-dnc^{+} flies exhibit normal olfactory acuity for octanol (Oct, $F_{4/42} = 1.53$, p = 0.21, n ≥ 8) and methylcyclohexanol (MCH, $F_{4/42} = 0.43$, p = 0.78, n ≥ 8) as well as normal shock response ($F_{4/42} = 1.02$, p = 0.41, n ≥ 8). Inducing Trh knockdown by Trh^{RNAi1} or Trh^{RNAi2} in $Gal80^{ts}$;GH298 and $Gal80^{ts}$;VT026326- flies resulted in normal olfactory acuity for octanol (Oct, $F_{4/34} = 0.38$, p = 0.904, n ≥ 7) and methylcyclohexanol (MCH, $F_{4/34} = 0.36$, p = 0.91, n ≥ 7) as well as normal shock response ($F_{4/34} = 0.92$, p = 0.49, n ≥ 8). Inducing 5HT-2A knockdown in the SPN of $Gal80^{ts}$;NP0047 or $tubGal80^{ts}$;NP2758 expressing UAS-5HT-$2A^{RNAi1}$ or UAS-5HT-$2A^{RNAi2}$ flies resulted in normal olfactory acuity for octanol (Oct) when using RNAi1 ($F_{4/44} = 0.54$, p = 0.58, n ≥ 7) or RNAi2 ($F_{4/40} = 0.5$, p = 0.61, n ≥ 7), normal olfactory acuity for methylcyclohexanol (MCH) when using RNAi1 ($F_{2/23} = 0.51$, p = 0.6, n ≥ 7) or RNAi2 ($F_{4/42}$ = 0.36, p = 0.69, n ≥ 7), and normal shock response when using RNAi1 ($F_{4/42} = 0.92$, p = 0.49, n ≥ 7) or RNAi2 ($F_{2/40} = 3.1$, p = 0.061, n ≥ 7). Data are presented as mean ± SEM. Statistical tests were performed using one-way ANOVA. No significant difference was found in a Newman-Keuls post-hoc comparison with each parental control.
